# Global, regional, and national burden of disease for high BMI-related ischemic stroke in people aged 70 and older: trend analysis from 1990 to 2021 and projections for 2044

**DOI:** 10.3389/fneur.2026.1794665

**Published:** 2026-05-22

**Authors:** Zhendong Huang, Yu Min, Guowen Zhang, Ji Li

**Affiliations:** 1 Department of Rehabilitation Medicine, The Affiliated Panyu Central Hospital, Guangzhou Medical University, Guangzhou, China; 2 School of Laboratory Medicine and Biotechnology, Southern Medical University, Guangzhou, China; 3 Northeastern University, Shenyang, China

**Keywords:** BMI, disability-adjusted life years, GBD 2021, ischemic stroke, trend projections

## Abstract

**Background:**

Ischemic stroke (IS) remains a leading cause of death and long-term disability worldwide. In older adults, metabolic risk factors have become increasingly significant, with high body mass index (BMI) contributing notably to the burden of IS. Although recent GBD-based studies have described the burden of high BMI-attributable ischemic stroke (HB-IS) across broader populations, they do not fully capture heterogeneity within adults aged ≥ 70 years. In this age group, cross-national inequalities, within-elderly age gradients, SDI- and sex-specific temporal patterns, and future trajectories under population aging remain insufficiently characterized.

**Methods:**

Using Global Burden of Disease 2021 estimates for 1990–2021, we quantified HB-IS deaths, disability-adjusted life years (DALYs), and population-attributable fractions (PAFs) among adults aged ≥ 70 years by sex, age, country, and socio-demographic index (SDI). Temporal trends were assessed using segmented log-linear regression with BIC-selected breakpoints, and period differences were summarized using period rate ratios (PRRs). Changes in deaths were decomposed using a Shapley-based approach into population growth, population aging, and rate change. Deaths were projected for 2022–2044 using empirical age-specific log-linear extrapolation with hindcasting-based validation.

**Results:**

In 2021, death rates ranged from 3.93 to 124.33 per 100,000 and DALY rates from 52.32 to 2085.76 per 100,000; PAFs was 0.53%−10.39% for deaths and 0.64%−11.34% for DALYs. DALY rates declined globally, driven by high-SDI regions, whereas low and low-middle SDI regions rose or stagnated. Within the ≥70-year population, DALY rates generally increased with age, and trends were generally less favorable in lower-SDI settings and among males. Deaths increased from 55,302 in 1990 to 83,007 in 2021. Under continuation of recent trends, model consistent forecasts projected deaths to reach 104,638 by 2044 despite falling rates, with growth increasingly concentrated in those aged ≥80 years.

**Conclusion:**

HB-IS among adults aged ≥ 70 years shows marked geographic, SDI-, sex-, and age-related disparities; despite falling rates, population growth and accelerated aging are likely to increase the absolute burden, supporting SDI-tailored and age-/sex-sensitive prevention and care.

## 1 Introduction

Ischemic stroke (IS) remains a leading cause of death and long-term disability worldwide, imposing a substantial public health burden through high mortality and persistent functional impairment (1). Advancing age is among the strongest risk factors for IS; age-related vascular remodeling, endothelial dysfunction, and heightened cardiometabolic vulnerability collectively increase stroke susceptibility and worsen outcomes in older adults (2, 3). Nevertheless, existing GBD-based studies have primarily examined high BMI-attributable ischemic stroke (HB-IS) or high BMI-attributable stroke and its subtypes across the full age spectrum (4, 5), whereas older-adult analyses have focused on overall ischemic stroke in broader populations, such as adults aged ≥60 years (6). Consequently, HB-IS among adults aged ≥70 years remains incompletely characterized. In particular, limited evidence is available on how HB-IS burden varies across 5-year age bands within this age-restricted population, whether SDI- and sex-specific temporal patterns diverge after restriction to ≥70 years, and how population growth and population aging may jointly shape future HB-IS burden in this subgroup. By focusing specifically on HB-IS among adults aged ≥70 years and integrating within-elderly age patterns, SDI-stratified trend analyses, decomposition of change, and model-based projections to 2044, the present study aims to provide evidence directly relevant to aging-focused prevention and health-system planning. Supplementary Table S1 provides a direct comparison between the present study and the most relevant prior all-age GBD analysis (4), further clarifying the incremental contribution of this age-restricted analysis.

The burden of IS is closely intertwined with socioeconomic development and healthcare system capacity (7). Regions with limited access to prevention, acute stroke care, and rehabilitation services often experience worse outcomes (8, 9), whereas countries undergoing rapid lifestyle transitions may face increasing metabolic risks despite improvements in care (10, 11). Among these risks, high BMI has become a prominent and potentially preventable contributor to stroke burden worldwide (11, 12). High BMI increases IS risk primarily via downstream cardiometabolic disturbances—especially elevated blood pressure (13), dyslipidaemia, insulin resistance, chronic inflammation, and endothelial/prothrombotic dysfunction. Clinical guidance also supports weight management as part of comprehensive vascular risk reduction after stroke. Therefore, it is essential to quantify between-country and SDI-stratified disparities, detect temporal inflection points in trends, characterize age- and sex-specific patterns, and anticipate future burden of HB-IS among adults aged ≥70 years to inform targeted interventions and resource allocation.

The GBD study is a comprehensive epidemiological initiative that quantifies health loss from diseases, injuries, and risk factors across countries and over time (14). Using the GBD 2021 estimates, this study aimed to: (i) quantify national variation in 2021 deaths, DALYs, and population-attributable fractions (PAFs) of IS attributable to high BMI among adults aged ≥70 years; (ii) assess temporal trends from 1990 to 2021 (including changes in trend segments where applicable) across SDI strata; (iii) examine heterogeneity by age, sex, and SDI; and (iv) project the burden to 2044 and decompose changes to quantify the relative contributions of demographic change and epidemiological change.

## 2 Methods

### 2.1 Data source and case definition

Data for this study were obtained from the GBD 2021 study through the IHME GBD Results tool (GHDx/IHME). Following the standardized GBD framework for data compilation, cause modeling, and comparative risk assessment, we extracted estimates of deaths and DALYs attributable to HB-IS among people aged ≥70 years from 1990 to 2021, together with corresponding rates (per 100,000 population) (15–17). Estimates were stratified by sex (male, female, and both sexes) and by 5-year age groups (70–74, 75–79, 80–84, 85–89, 90–94, and ≥95 years). The GBD 2021 provided 95% uncertainty intervals (UIs) (16).

In GBD 2021, IS is defined using an established cause mapping based on the International Classification of Diseases (ICD) coding system and corresponding ICD-9 codes; IS typically corresponds to ICD-10 code I63. High BMI is treated as a risk factor in the GBD comparative risk assessment; the attributable burden represents the counterfactual reduction in IS burden that would occur if BMI were shifted to the theoretical minimum risk exposure level used in the GBD.

### 2.2 Socio-demographic index

SDI is a composite indicator developed by IHME to summarize a location's level of socioeconomic development in relation to health outcomes. SDI is calculated as the geometric mean of three normalized components: lag-distributed income per capita, average educational attainment among individuals aged ≥15 years, and total fertility rate among females aged < 25 years. SDI values range from 0 (lowest development) to 1 (highest development). In line with the GBD reporting, countries and territories were grouped into five SDI quintiles (low, low-middle, middle, high-middle, and high) to facilitate comparisons across development levels.

### 2.3 Burden measures and rates

We assessed the burden of HB-IS using two standard GBD measures: deaths and DALYs. DALYs are the sum of years of life lost (YLLs) due to premature mortality and years lived with disability (YLDs), thereby capturing both fatal and non-fatal health loss. For the population aged ≥70 years, we used the GBD-provided age-specific rates (per 100,000) for each 5-year age group, as well as aggregated rates for the overall ≥70-year population (computed as total deaths or DALYs divided by total population aged ≥70 years, multiplied by 100,000) (17). All rates are reported per 100,000 population unless otherwise specified.

### 2.4 Segmented log-linear regression

For each stratum defined by sex and SDI, annual HB-IS rates were modeled using segmented log-linear regression. Candidate piecewise linear models with 1–6 segments were fitted to the natural logarithm of the annual rate using the pwlf package. Breakpoints were constrained to integer years within the observed range (1990–2021), with a minimum segment length of 5 years. The final model was selected using the Bayesian information criterion (BIC). APCs for each segment were calculated as (exp(β) – 1) × 100 from log-linear slopes estimated within each segment, and statistical inference was based on standard errors robust to heteroskedasticity and autocorrelation (HAC, maxlag = 1). The overall average annual percent change (AAPC) for 1990–2021 was estimated from a separate log-linear model fitted over the full period within each stratum rather than as a weighted average of APCs for individual segments. The final figure displays joinpoints selected by BIC and overall AAPCs, whereas APCs for individual segments are reported in Supplementary Table S4.

### 2.5 APC analysis by age group

APCs by age group were estimated for age bands of 5 years each (70–74, 75–79, 80–84, 85–89, 90–94, and ≥95 years) within each stratum defined by SDI and sex using log-linear regression of annual DALY rates from 1990 to 2021. Specifically, ordinary least squares models were fitted to the natural logarithm of the annual rate, and standard errors robust to heteroskedasticity and autocorrelation were estimated using a HAC covariance estimator (maxlag = 1). APCs were calculated as (exp(β) – 1) × 100. The 95% confidence interval was derived from β ± 1.96 × SE and then transformed back to the APC scale. These estimates are summarized in Supplementary Table S5 and visualized in Supplementary Figure S1.

### 2.6 Period rate ratio (PRR) calculation

Calendar years were grouped into consecutive periods of 5 years (1990–1994, 1995–1999,..., 2020–2021). For each stratum defined by measure, location, and sex, the rate for each period was defined as the mean of the annual rates within that period. The period rate ratio (PRR) was then calculated as the mean rate for each period divided by the corresponding mean rate in the reference period (1990–1994). Lower and upper bounds were summarized descriptively by dividing the mean lower and upper estimates for each period by the corresponding mean rate in the reference period. PRRs were used as descriptive summaries of relative burden across calendar periods rather than as estimates from a formal age–period–cohort model.

### 2.7 Forecasting, validation, and sensitivity analysis

Forecasts for 2022–2044 were generated using an empirical log-linear extrapolation of rates by age group rather than a formal age–period–cohort or Bayesian framework. Populations by age group were reconstructed from GBD counts and rates, and separate log-linear models were fitted to series of rates and populations by age group over 2000–2021. Projected deaths were obtained by combining projected rates and projected populations within each stratum defined by age and sex and aggregating across strata. To maintain internal consistency between forecasting and decomposition, the primary 2021 baseline for the 2021–2044 decomposition was anchored to the fitted estimate from the projection model. As a sensitivity analysis, the decomposition was repeated using the descriptive GBD 2021 estimates by age group as the baseline while keeping the projected 2044 populations and rates by age group unchanged. Model validation was performed using hindcasting, in which models were trained on 2000–2014 and evaluated against observed data from 2015–2021. Predictive performance was summarized using mean absolute percentage error (MAPE), mean absolute error (MAE), root mean square error (RMSE), and the proportion of observed values in the test period covered by the 95% prediction interval from the model. As an additional robustness check, the primary log-linear forecast for the globally aggregated death rate series among adults aged ≥70 years was compared with an unobserved components model (UCM) with a local linear trend and an ARIMA model selected by Akaike information criterion (AIC). Differences from the primary forecast were summarized using the mean absolute percentage difference (MAPD) over 2022–2044 and the percentage difference in 2044.

### 2.8 Statistical analysis

Temporal trends in annual HB-IS rates were assessed using segmented log-linear regression stratified by sex and SDI level. For each stratum, candidate piecewise linear models with 1 to 6 segments were fitted to the natural logarithm of the annual rate using the pwlf package. Breakpoints were constrained to integer years within 1990–2021, with a minimum segment length of 5 years, and the final model was selected using the Bayesian information criterion (BIC). Segment-specific annual percent changes (APCs) were calculated as (exp(β) – 1) × 100 using heteroskedasticity- and autocorrelation-consistent standard errors (HAC, maxlag = 1), and the overall average annual percent change (AAPC) for 1990–2021 was estimated from a separate full-period log-linear model. Age-specific APCs for 5-year age bands (70–74 to ≥95 years) were estimated using analogous log-linear models with HAC standard errors and are summarized in Supplementary Table S5 and Supplementary Figure S1. Calendar years were grouped into 5-year periods (1990–1994, 1995–1999,..., 2020–2021), and period rate ratios (PRRs) were calculated as the mean rate in each period divided by the corresponding mean rate in the reference period (1990–1994) within the same measure, location, and sex stratum. PRRs were used as descriptive summaries of relative burden across periods rather than as estimates from a formal age–period–cohort model. Accordingly, APC/AAPC quantify the pace of annual change, whereas PRR summarizes the relative level of burden compared with the baseline period. Age-specific patterns were described using cross-sectional age-specific DALY rates across 5-year age groups. Segment-specific APCs are reported in Supplementary Table S4, and PRRs are reported in Supplementary Table S6.

Changes in deaths were decomposed into the contributions of population growth, population aging, and age-specific rate change using a Shapley-based approach to ensure additivity and order invariance. For forecasting (2022–2044), we used an empirical age-specific log-linear extrapolation rather than a formal age–period–cohort or Bayesian framework. Age-specific populations were reconstructed from GBD counts and rates, and separate log-linear models were fitted to age-specific rate and population series over 2000–2021. Projected deaths were obtained by combining projected rates and projected populations within each age–sex stratum and aggregating across strata. To maintain internal consistency between forecasting and decomposition, the primary 2021 baseline for the 2021–2044 decomposition was anchored to the model-fitted estimate from the projection model. As a sensitivity analysis, we repeated the 2021–2044 decomposition using the descriptive GBD 2021 age-specific estimates as the baseline while keeping the projected 2044 age-specific populations and rates unchanged. Model performance was assessed by hindcasting, in which models were trained on 2000–2014 and evaluated against observed data from 2015–2021, using mean absolute percentage error (MAPE), mean absolute error (MAE), root mean square error (RMSE), and 95% prediction-interval coverage. For the global aggregated death-rate series among adults aged ≥70 years, projection robustness was further examined by comparing the primary log-linear forecast with an unobserved components model (UCM) and an Akaike information criterion (AIC)-selected ARIMA model, summarizing differences using the mean absolute percentage difference (MAPD) over 2022–2044 and the percentage difference in 2044. Hindcast performance metrics and robustness comparisons are provided in Supplementary Tables S7, S8. For aggregated estimates (both sexes and ≥70 years), rates were computed as population-weighted averages of stratum-specific rates (equivalently, as total counts divided by total population). Because forecast uncertainty increases with the projection horizon and may vary across settings, projected values were interpreted as scenario-based estimates under continuation of recent trends rather than deterministic predictions.

All analyses were performed using Python 3.10.0 (pandas, NumPy, statsmodels, pwlf, and Matplotlib), and reporting followed GATHER principles (18).

## 3 Results

### 3.1 HB-IS deaths and DALYs global burden in 2021

**Figure 1:**
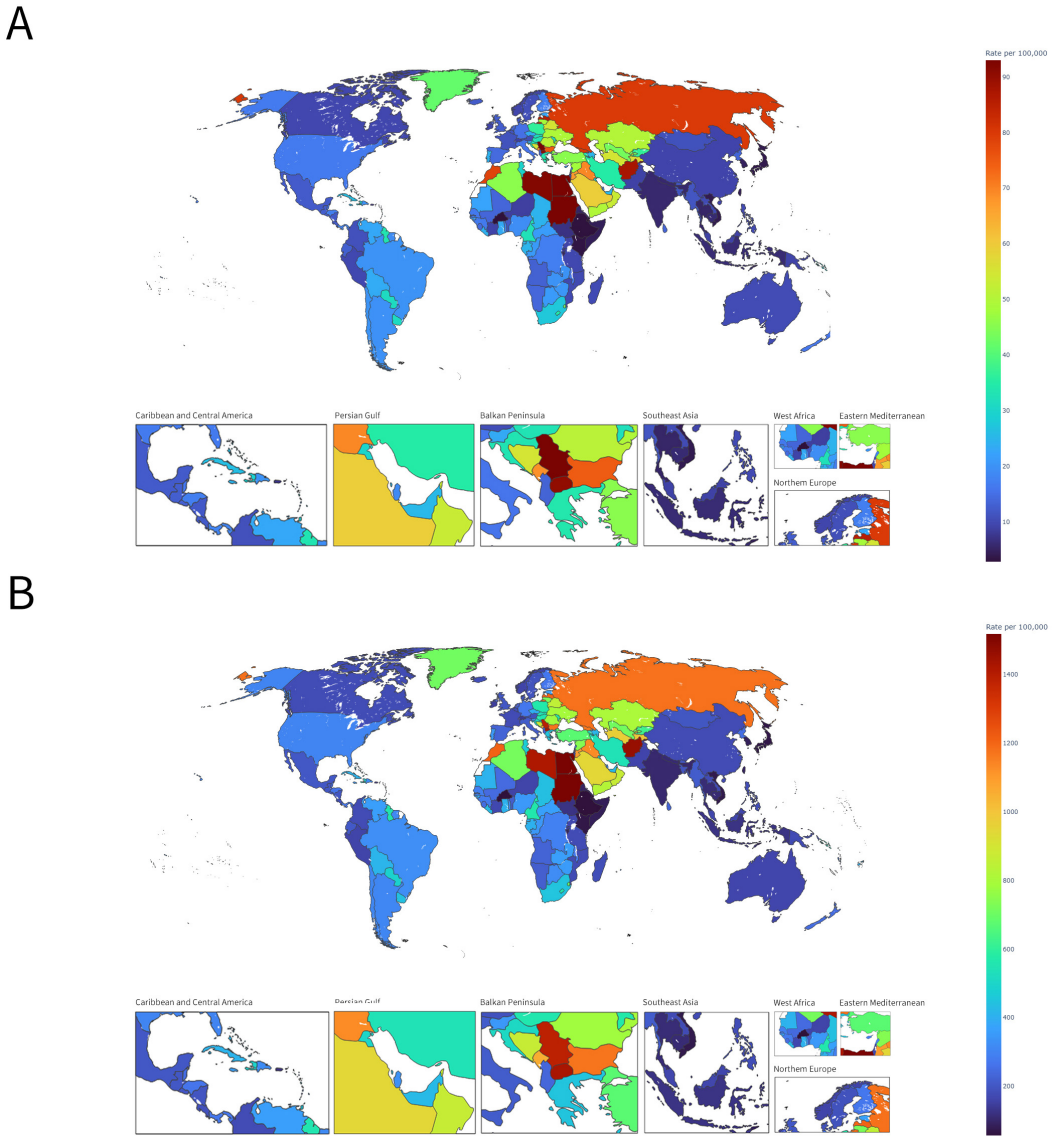
Global distribution of HB-IS burden in adults aged ≥70 years, 2021. **(A)** Death rate, deaths per 100,000 population; **(B)** DALY rate, DALYs per 100,000 population.

In 2021, the burden of HB-IS among people aged ≥70 years showed marked geographic heterogeneity across 204 countries and territories (Figure 1; Table 1). Death rates varied widely, with particularly high levels observed in North Africa and the Middle East, exemplified by Egypt (124.330 per 100,000), and elevated burdens also seen in parts of southern Africa (e.g., Eswatini, 43.318) and the Persian Gulf (e.g., United Arab Emirates, 25.408; Qatar, 18.838). In contrast, several settings in East Asia had comparatively low rates, such as Japan (3.934), while other countries showed moderate burdens, including the United States of America (16.514) and Mongolia (10.945) (Figure 1A). A similar geographic pattern was observed for DALY rates, ranging from lower levels in Ethiopia (52.324) and Japan (69.765) to substantially higher levels in Egypt (2085.762), with notable burdens also present in Eswatini (694.755) and Zimbabwe (355.930) (Figure 1B). Overall, these findings indicate substantial cross-national disparities in both fatal and non-fatal health loss attributable to HB-IS in the ≥70-year population.

**Table 1:** Death and DALY rates of HB-IS among individuals aged 70+ in selected countries in 2021.

Country	Death rate (per 100,000)	DALY rate (per 100,000)
Egypt	124.330 (21.211–245.313)	2085.762 (369.375–4127.410)
Sudan	93.839 (15.425–205.440)	1520.960 (251.164–3303.125)
Serbia	93.102 (14.792–196.081)	1386.443 (227.810–2854.311)
North Macedonia	90.346 (14.474–195.007)	1429.714 (234.409–3009.444)
Latvia	85.368 (15.060–178.737)	1202.216 (219.141–2424.206)
Georgia	79.996 (12.646–173.904)	1166.392 (192.138–2453.970)
Nauru	79.695 (12.784–170.504)	1550.458 (252.942–3228.841)
Morocco	78.395 (12.336–173.138)	1206.134 (194.424–2600.848)
Tuvalu	77.955 (13.642–177.545)	1357.610 (241.797–2973.252)
Brazil	20.135 (3.510–39.779)	325.291 (57.219–640.014)
United States of America	16.514 (3.094–33.502)	302.556 (56.044–616.478)
China	9.254 (1.719–20.263)	175.873 (32.445–379.091)
India	5.755 (0.897–13.099)	104.411 (16.512–228.489)
Japan	3.934 (0.635–8.631)	69.765 (11.208–150.394)

**Figure 2:**
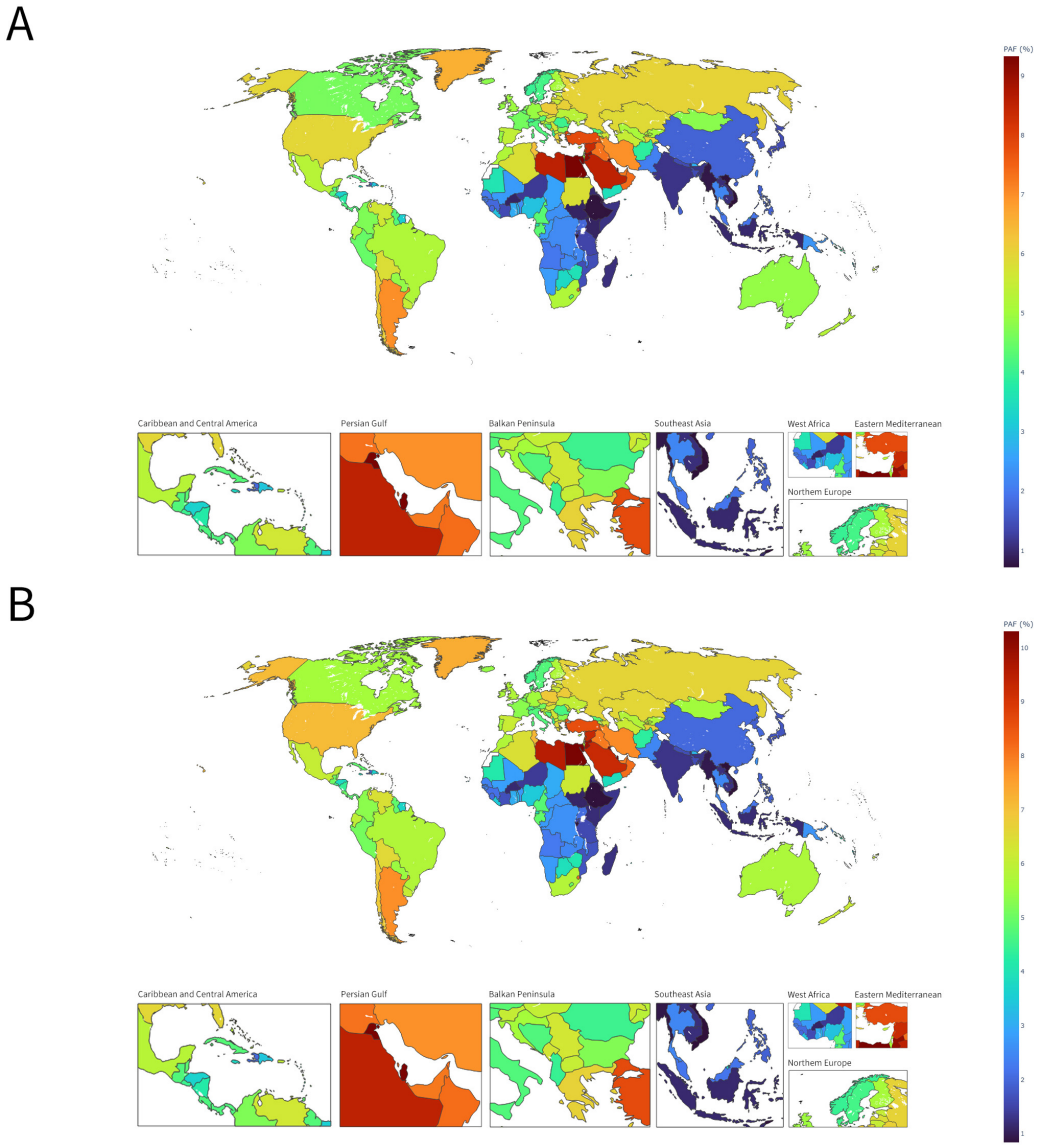
Global distribution of the population-attributable fraction (PAFs) of HB-IS burden in adults aged ≥70 years, 2021. **(A)** PAFs for deaths. **(B)** PAFs for DALYs.

In 2021, the population-attributable fraction (PAFs) of HB-IS among adults aged ≥70 years varied markedly across 204 countries and territories (Figure 2). PAFs for deaths ranged from 0.53% in Viet Nam to 10.39% in Egypt, with the highest attributable fractions clustering in North Africa and the Middle East/Persian Gulf (e.g., Kuwait 9.90%, Qatar 9.34%, Jordan 9.04%, Palestine 8.76%, Saudi Arabia 8.53%) and in some Pacific island settings (e.g., Nauru 8.63%) (Figure 2A). A similar spatial pattern was observed for DALYs, with PAFs spanning 0.64% (Viet Nam) to 11.34% (Egypt), and consistently high values in Kuwait (11.20%), Qatar (10.33%), Jordan (9.88%), Palestine (9.65%), and Nauru (9.58%) (Figure 2B). In contrast, many countries in sub-Saharan Africa and parts of Southeast Asia showed low attributable fractions (typically < 2%) for both outcomes. Overall, DALYs PAFs were slightly higher than death PAFs on average (4.83 vs. 4.26%, +0.58 percentage points), indicating a somewhat greater relative contribution of high BMI to non-fatal disability than to mortality in this older population.

### 3.2 Temporal trends in HB-IS DALY rates by SDI level (1990–2021)

**Figure 3:**
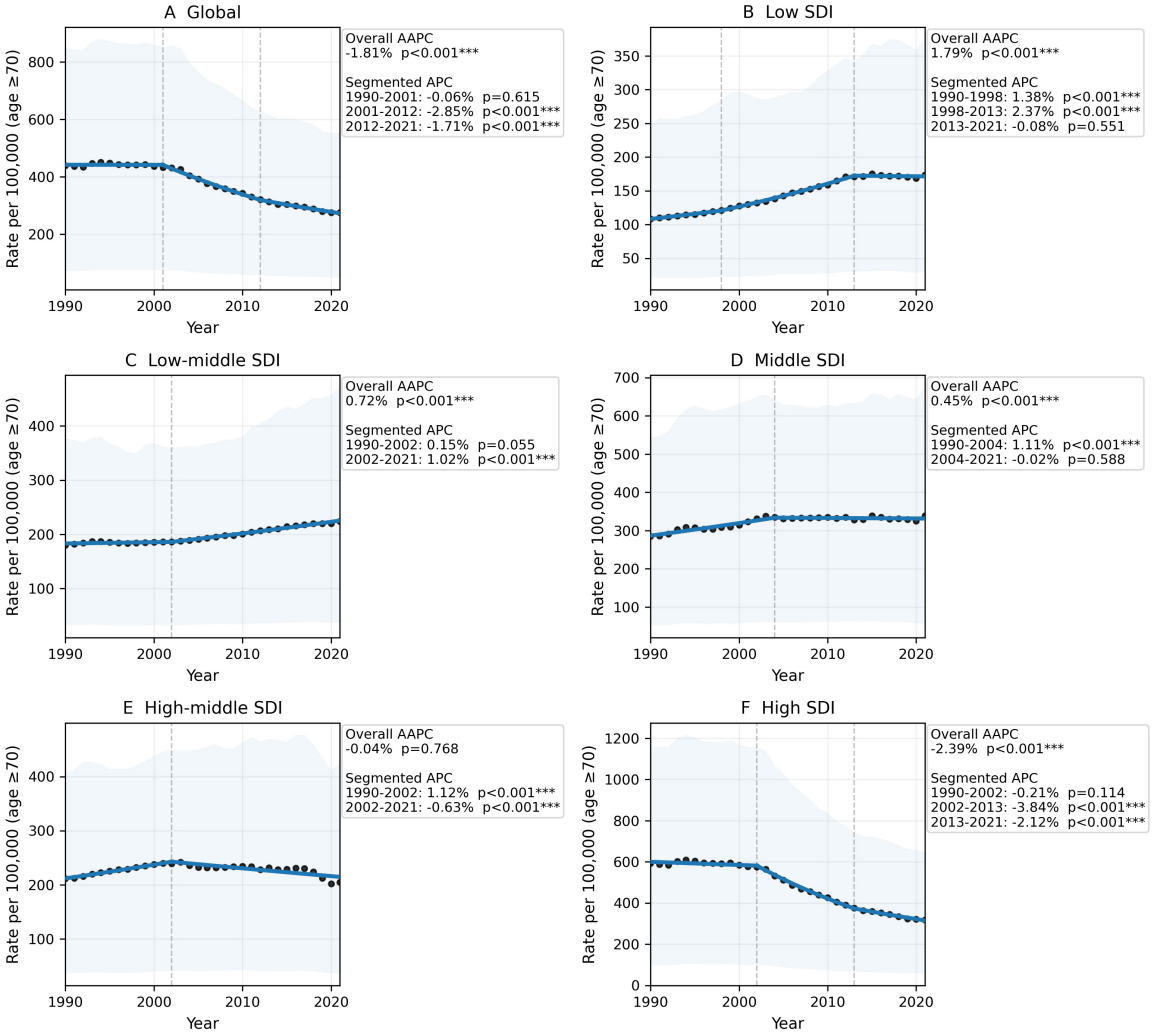
Temporal trends in HB-IS DALY rates among adults aged ≥70 years by SDI level, 1990–2021. **(A)** Global, **(B)** Low SDI, **(C)** Low-middle SDI, **(D)** Middle SDI, **(E)** High-middle SDI, and **(F)** High SDI. Panels show annual point estimates with GBD 95% uncertainty ribbons, BIC-selected segmented log-linear fits, estimated joinpoints, and overall AAPCs; segment-specific APCs are reported in Supplementary Table S4.

Across SDI levels, HB-IS DALY rates among adults aged ≥70 years showed distinct temporal patterns from 1990 to 2021 (Figures 3A–F). Globally, rates declined overall (AAPC −1.81%), shifting from stability in 1990–2001 (APC −0.06%) to sustained decreases thereafter (2001–2012 APC −2.85%; 2012–2021 APC −1.71%). High-middle SDI was broadly flat overall (AAPC−0.04%), rising before 2002 (APC 1.12%) and declining afterwards (APC−0.63%). In contrast, Low SDI increased through 2013 (APC 1.38% and 2.37%) before leveling off in 2013–2021 (APC −0.08%), while Low-middle SDI rose mainly after 2002 (APC 1.02%). Middle SDI increased early (APC 1.11%) and then plateaued after 2004 (APC −0.02%). Overall, the global decline was driven largely by higher-SDI settings, whereas persistent increases or stagnation in low and low-middle SDI regions suggest continued pressure and widening inequalities in the HB-IS DALYs burden in lower-resource settings.

### 3.3 Period-specific rate ratios and age-specific DALY-rate patterns across SDI levels

**Figure 4:**
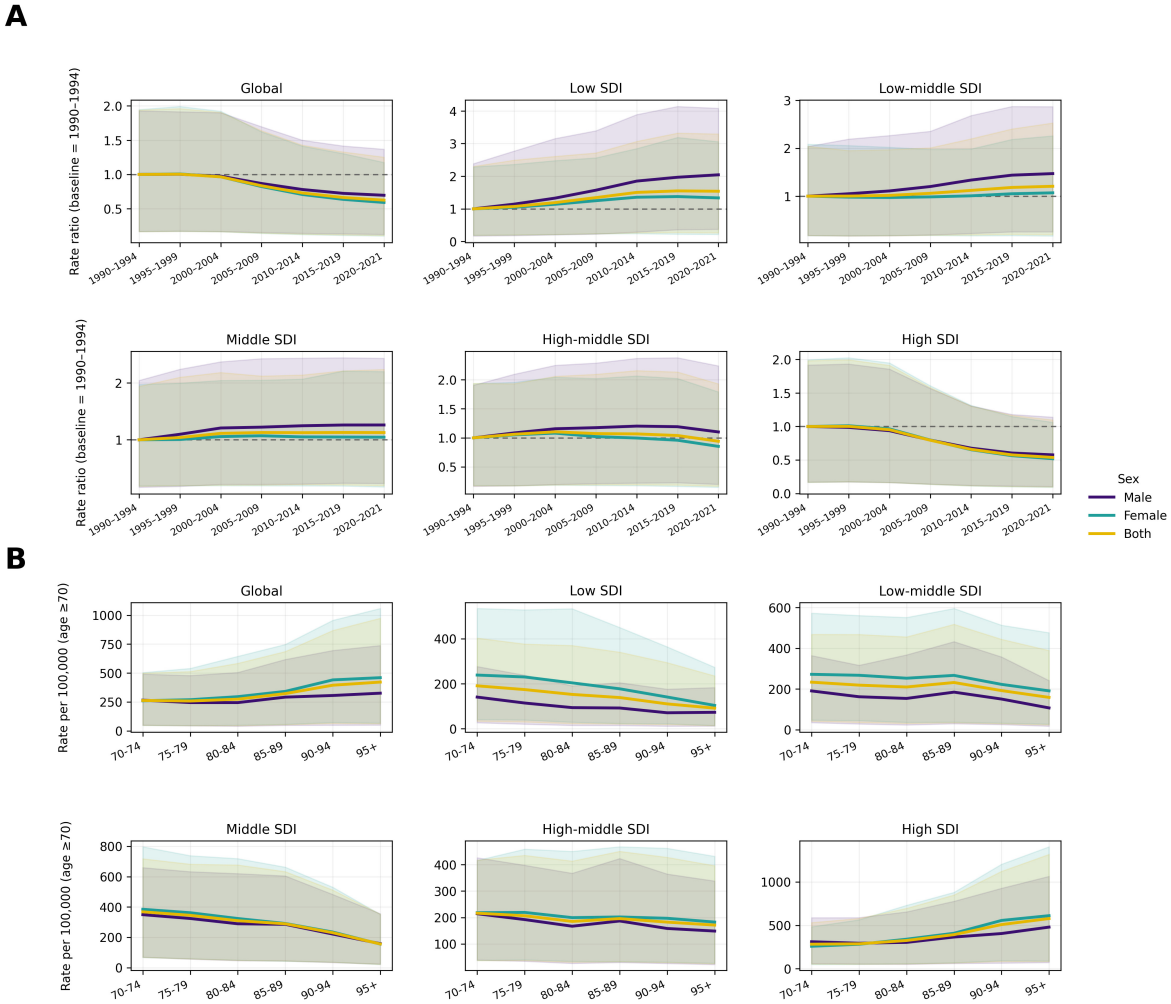
Period-specific rate ratios and age-specific DALY-rate patterns of HB-IS among adults aged ≥70 years by SDI level, 1990–2021. **(A)** PRRs relative to the baseline period (1990–1994), calculated as the mean rate in each 5-year period divided by the corresponding mean rate in 1990–1994 within the same stratum. **(B)** Age-specific DALY rates across 5-year age groups (70–74 to ≥95 years), stratified by sex.

Period-specific PRR patterns (Figure 4A) revealed clear SDI-stratified divergence in HB-IS DALY rate ratios over time. Globally, PRRs declined across successive periods, but the direction and magnitude varied by socioeconomic context. High SDI settings showed the most pronounced and sustained reductions, while high-middle SDI exhibited relatively modest change. In contrast, low, low-middle, and middle SDI regions displayed overall upward shifts in PRRs, indicating a growing relative burden in lower-resource settings. Sex differences were evident across most SDI strata, with males generally showing less favorable period trends than females, particularly in low SDI regions.

Age specific patterns in 2021 (Figure 4B) demonstrated heterogeneous within-elderly gradients across SDI levels. Globally, DALY rates increased with advancing age, with differences between males and females becoming more apparent at older ages. High SDI regions showed the steepest age-related increase, especially in the oldest age groups, reflecting a strong accumulation of burden with age. High-middle SDI also exhibited an age-related rise, though less pronounced than in high SDI settings. By contrast, low and middle SDI regions showed flatter or even declining patterns at the oldest ages, suggesting a different age distribution of burden within the ≥70-year population. Overall, the combination of period- and age-specific patterns highlights substantial socioeconomic and sex-related disparities in HB-IS DALYs burden among older adults. Consistent with these cross-sectional age gradients, age-specific APC analyses indicated that temporal changes within the ≥70 population differed by age band and SDI level, with generally less favorable trends in lower-SDI settings and among males (Supplementary Figure S1).

### 3.4 Projected global trends in HB-IS burden for individuals aged 70 and above, 2021–2044

**Figure 5:**
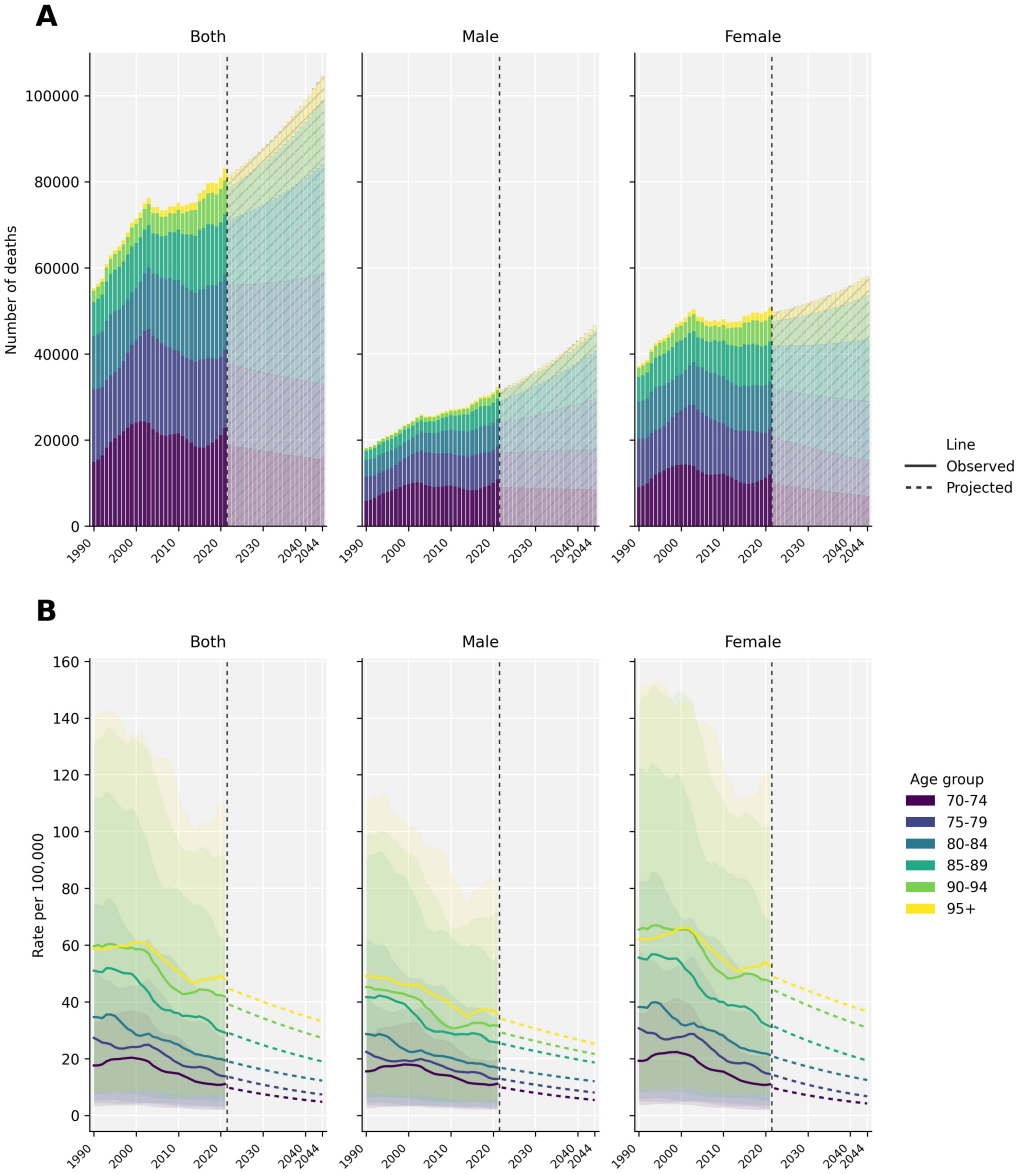
Projected HB-IS deaths and death rates among adults aged ≥70 years globally, 2021–2044. **(A)** Projected number of deaths. **(B)** Projected death rates per 100,000 population.

In the global forecasts for HB-IS among adults aged ≥70 years (Figure 5), the absolute number of deaths is projected to increase despite continued declines in death rates. As shown in Figure 5A, total deaths (both sexes) rise from approximately 80,800 in 2021 to approximately 104,600 in 2044, with the projected growth increasingly concentrated in the oldest age groups (≥80 years)—particularly 80–84, 85–89, and 90–94 years—while deaths in 70–74 and 75–79 years are projected to plateau or decline. Sex-specific patterns indicate that females contribute more deaths throughout the period, but the relative increase is steeper in males, narrowing the sex gap in projected counts.

In contrast, age-specific death rates per 100,000 decline across all age bands (Figure 5B). Rates retain a strong age gradient (highest in ≥95 years) but are projected to fall from 16.35 per 100,000 in 2021 to 10.71 per 100,000 in 2044 for both sexes combined. Overall, the forecasts highlight a classic “rates down, numbers up” pattern, where risk reduction is outweighed by demographic expansion and a shift toward older age structure within the ≥70-year population. These forecasts should be interpreted as scenario-based estimates under continuation of recent trends, with greater uncertainty toward the end of the forecast horizon and potentially larger variability across specific regions.

### 3.5 Drivers of changes in HB-IS deaths: three-factor decomposition

**Figure 6:**
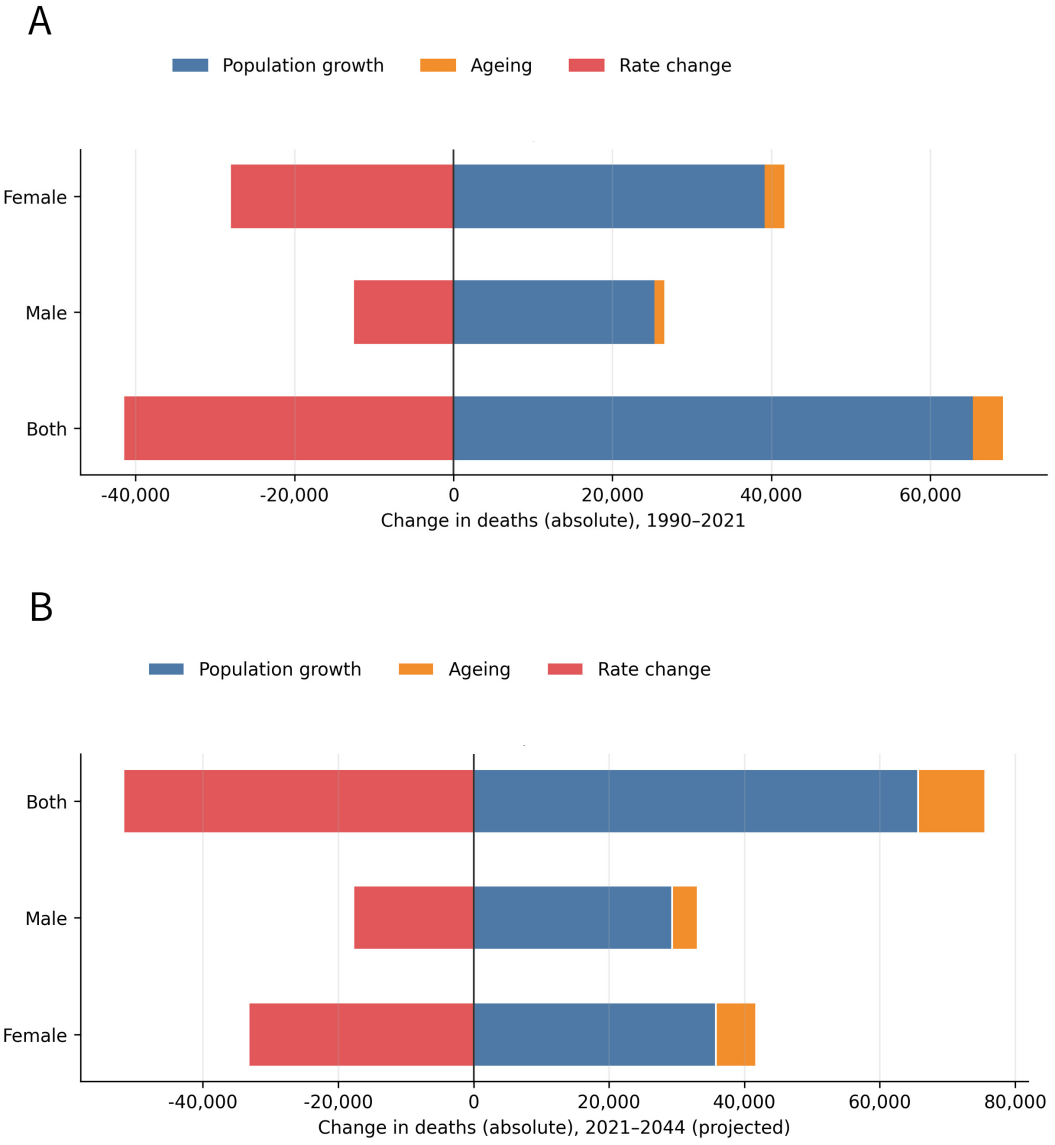
Three-factor decomposition of changes in HB-IS deaths among adults aged ≥70 years at the global level. **(A)** Decomposition of the absolute change in deaths from 1990 to 2021 into population growth, population aging (age structure), and rate change. **(B)** Decomposition of the projected absolute change in deaths from 2021 to 2044 using the same components, stratified by sex. The 2021 baseline used for the 2021–2044 decomposition is the model-fitted estimate (80,781), whereas the descriptive GBD 2021 estimate is 83,007.

The three-factor decomposition is shown in Figure 6. From 1990 to 2021, global HB-IS deaths among adults aged ≥70 years increased from 55,302 to 83,007 (+27,705) (Figure 6A). This net increase was mainly driven by population growth (+65,340) and, to a lesser extent, population aging (+3,805), while improvements in age-specific rates contributed a large negative offset (rate change −41,440), counteracting ~59.9% of the demographic-driven increase. Sex-stratified results showed larger demographic contributions in females than males, with a stronger offset from declining rates in females.

Using the model-consistent 2021 baseline, projections to 2044 indicate that deaths will continue to rise from 80,781 to 104,638 (+23,858) (Figure 6B), with population growth (+65,658) remaining the dominant driver and the aging contribution increasing markedly (+9,927). Although further reductions in rates (−51,728) are projected, they are insufficient to fully offset the combined effects of population growth and accelerated aging, particularly in females.

In a sensitivity analysis using the descriptive GBD 2021 baseline instead of the model-fitted 2021 baseline, the projected 2044 deaths remained unchanged at 104,638, whereas the net increase from 2021 to 2044 decreased from 23,858 to 21,632; the contributions of population growth, population aging, and rate change changed from (+65,658), (+9,927), and (−51,728) to (+65,218), (+11,579), and (−55,166), respectively (Supplementary Figure S2; Supplementary Tables S2, S3).

### 3.6 Model validation and robustness of HB-IS burden projections

**Figure 7:**
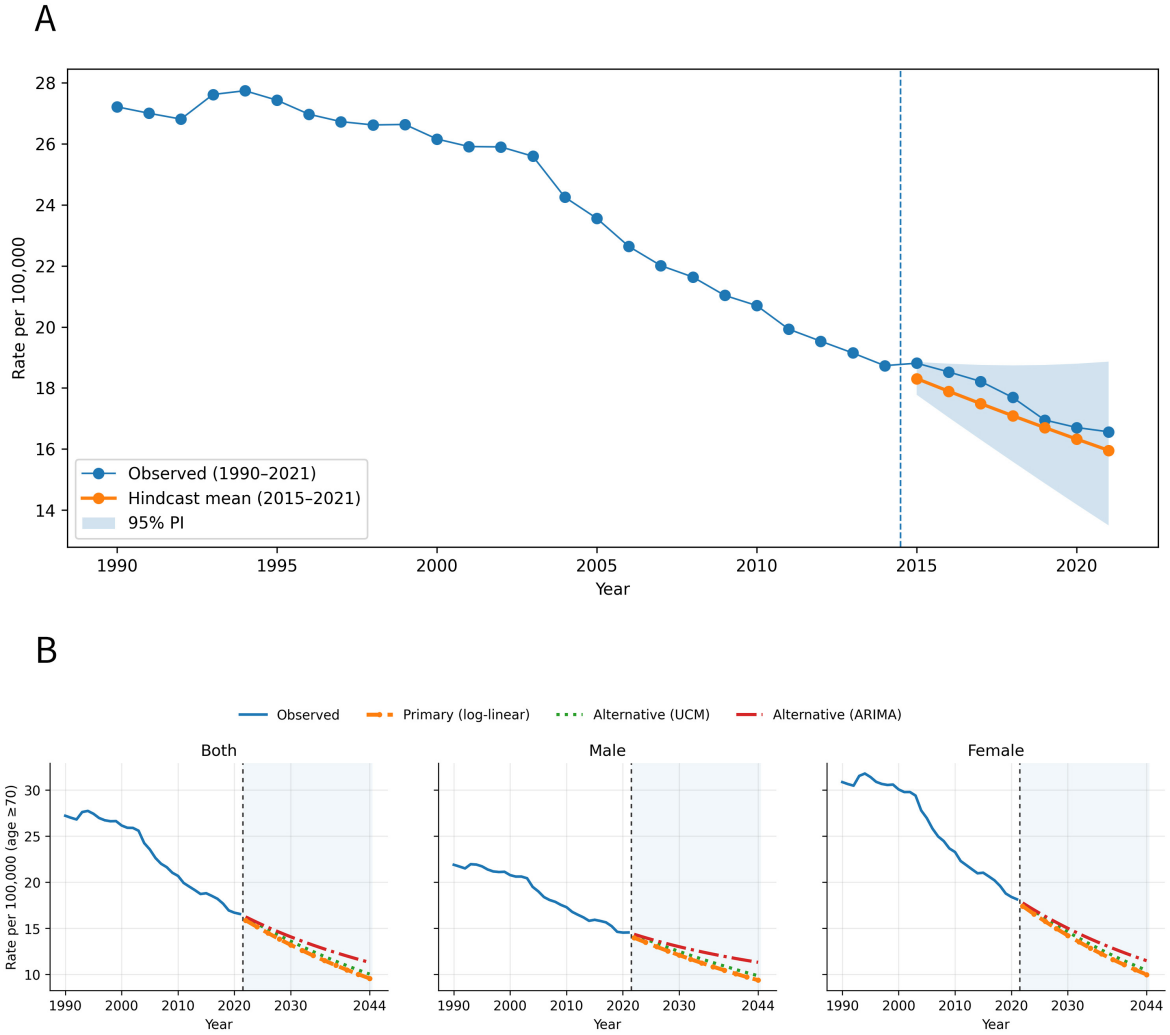
Hindcasting validation and multi-model robustness of projected HB-IS death rates among adults aged ≥70 years, global. **(A)** Hindcast performance for 2015–2021 (trained on 2000–2014) with 95% prediction intervals. **(B)** Comparison of projected trends to 2044 using the primary log-linear model vs. alternative UCM and ARIMA models, stratified by sex.

Model validation and robustness (Figures 7A, B). In hindcasting analyses (trained on 2000–2014 and tested on 2015–2021), the primary log-linear model closely reproduced the observed decline in HB-IS rates among adults aged ≥70 years. At the global level, prediction errors were low (MAPE 2.98% and RMSE 0.55 for death rates; MAPE 5.10% and RMSE 15.33 for DALY rates), and the observed values were fully covered by the 95% prediction interval. Across SDI strata, performance remained acceptable, with MAPE ranging from 0.75 to 7.58% for death rates and from 0.93% to 7.28% for DALY rates; 95% prediction-interval coverage varied by setting but was generally higher at the global level than in some lower-SDI strata. Robustness checks further showed that projected death-rate trajectories to 2044 were broadly consistent across alternative time-series specifications: compared with the primary forecast, the UCM yielded very similar projections (MAPD 3.10%−3.69%; 2044 rates ~4.52%−5.09% higher), whereas ARIMA produced higher 2044 rates but preserved the same overall declining trend (MAPD 7.63%−10.55%; 2044 rates ~15.00%−20.71% higher), supporting the stability of the main projection conclusions.

### 3.7 Socioeconomic heterogeneity in demographic and epidemiological drivers of HB-IS deaths (1990–2021)

**Figure 8:**
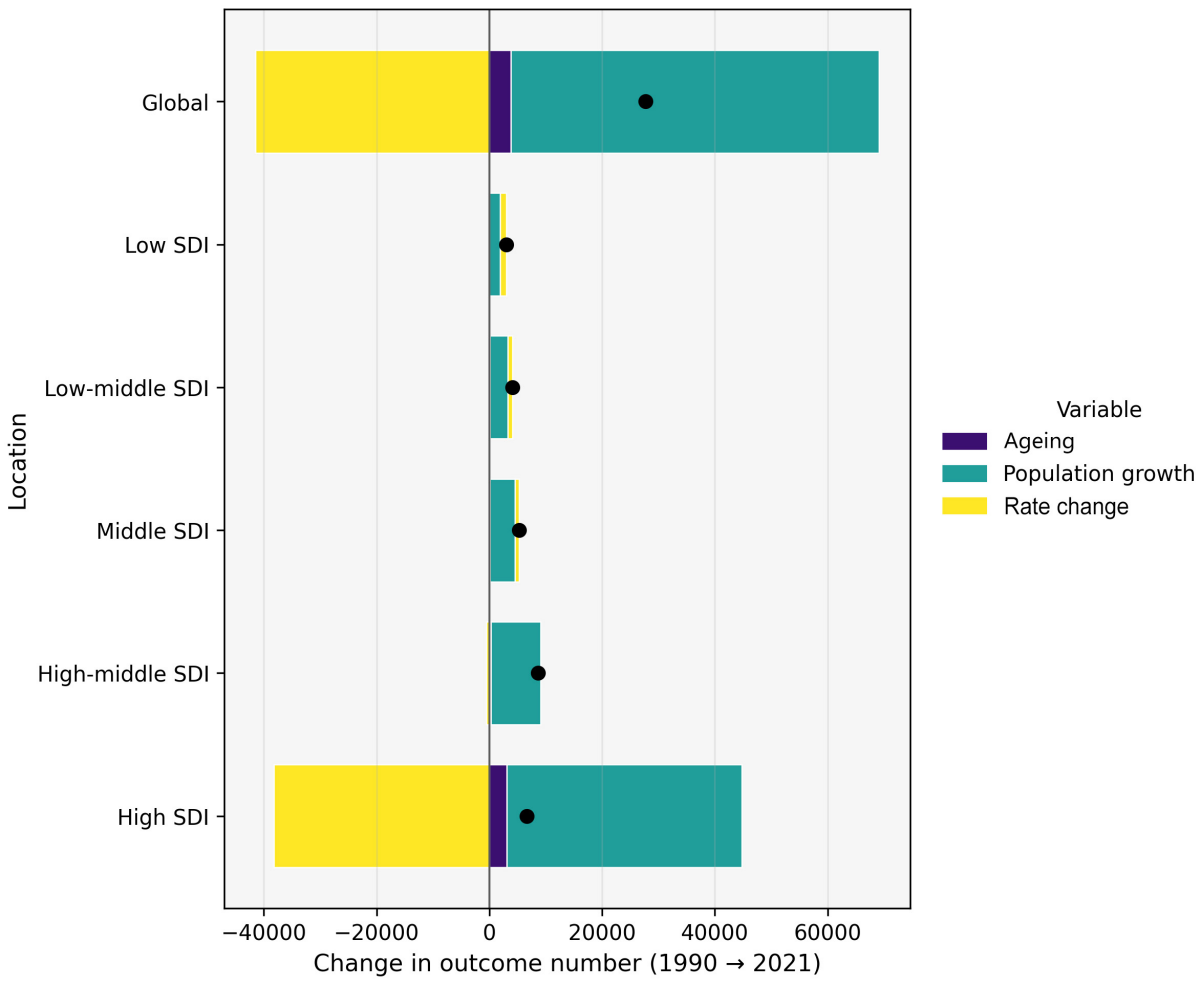
SDI-stratified three-factor decomposition of changes in HB-IS deaths among adults aged ≥70 years, 1990–2021. Bars show the contributions of population growth, population ageing, and age-specific rate change to the absolute change in deaths. Black dots indicate the total net change.

Figure 8 summarizes the drivers of changes in the absolute HB-IS burden across SDI strata. Decomposition results indicated that population growth and population aging jointly increased deaths in all SDI groups, while declines in age-specific rates offset part of these demographic pressures—most markedly in higher-SDI settings. In contrast, low and low-middle SDI regions experienced smaller offsetting effects from rate reductions, consistent with less favorable recent trends.

## 4 Discussion

Ischemic stroke remains a leading cause of death and long-term disability worldwide. Recent GBD-based all-age studies, including Guo et al. (4), have shown rising absolute deaths and DALYs attributable to high BMI despite declining age-standardized rates, and they have indicated that older adults bear a substantial share of this burden. By treating adults aged ≥70 years not merely as a subgroup across the life course but as the analytic population itself, our study extends this literature and reveals marked heterogeneity within the elderly population, including differences across 5-year age bands, divergence in temporal patterns by SDI and sex, a projected concentration of future deaths in those aged ≥80 years, and an increasing contribution of population aging to the absolute burden. Together, these findings indicate that HB-IS constitutes a meaningful but unequally distributed component of stroke burden in later life, consistent with broader evidence of geographic and sociodemographic disparities in stroke burden and risk factors (5). Using the GBD 2021 comparative risk assessment estimates, we characterized global, regional, and national patterns of HB-IS burden from 1990 to 2021, quantified socioeconomic and sex disparities via SDI-stratified trend and age/period analyses, and projected trajectories to 2044. Collectively, the results reveal a dual reality: encouraging declines in HB-IS rates in higher-SDI settings, contrasted with stagnation or increases in lower-SDI settings, alongside a projected rise in absolute deaths driven by demographic expansion and accelerated population aging. In 2021, HB-IS burden among older adults showed striking cross-national heterogeneity, with death and DALY rates varying by more than an order of magnitude across 204 countries and territories (19, 20). The highest death rates were concentrated in parts of North Africa and the Middle East (e.g., Egypt) and extended to several Eastern European and African settings, whereas much lower rates were observed in countries such as Japan. DALY rates mirrored the mortality distribution, indicating that areas with high fatal burden also experience substantial non-fatal health loss. The strong alignment between DALY and death rates, together with a mean DALY-to-death ratio of approximately 17.0 across 204 countries and territories, suggests that HB-IS in this age group is associated with substantial loss of healthy life per death. Notably, some small island settings showed above-average DALY-to-death ratios (e.g., Nauru, Tuvalu), which may reflect differences in case-fatality, survival with disability, stroke severity mix, access to acute care and rehabilitation, or population structure within the ≥70-year group. These findings emphasize that comparing mortality alone may underestimate the societal and healthcare impact of HB-IS in settings where survival is improving, but disability remains high.

Geographic disparities were further clarified by the population-attributable fraction (PAFs) attributable to high BMI. In 2021, PAFs ranged from very low values in several countries (e.g., Viet Nam) to high values in North Africa and the Middle East/Persian Gulf (e.g., Egypt, Kuwait, Qatar, Jordan, Palestine, Saudi Arabia) and certain Pacific island settings (e.g., Nauru) (21). The slightly higher mean PAFs for DALYs than for deaths suggests a somewhat larger attributable share of high BMI in non-fatal disability than in mortality in this older population. Within the GBD attribution framework, countries with high PAFs may warrant priority attention for integrated management of obesity-related and other cardiometabolic risks, whereas countries with low PAFs may require broader strategies addressing a wider mix of risk factors and health-system constraints.

Long-term trend analyses revealed a clear SDI-stratified divergence (22). Globally, HB-IS DALY rates among adults aged ≥70 years declined from 1990 to 2021, with a transition from relative stability in the 1990s to sustained decreases after 2001. However, this overall improvement was largely driven by higher-SDI settings. High-SDI regions showed the most pronounced and sustained reductions over time, consistent with stronger preventive strategies, better access to evidence-based therapies, and more mature stroke systems of care (23). In contrast, low and low-middle SDI regions exhibited persistent increases or stagnation, including rising PRRs, suggesting that the combined effects of epidemiologic transition (greater exposure to obesity and metabolic risk) and limited preventive/acute care capacity may be shifting HB-IS burden toward lower-resource settings (24, 25). The relatively flat pattern in high-middle SDI and plateauing in middle SDI likely reflects heterogeneous trajectories within these broad categories—some countries achieving rapid improvements in vascular risk management (26), while others experience increasing obesity prevalence, uneven access to care, and slower gains in stroke prevention (27). Possible mechanisms for regional and SDI disparities should be considered when interpreting these findings. First, secular increases in overweight and obesity have been substantial worldwide, with particularly high burdens in Pacific island settings and in parts of the Middle East and North Africa (28), which is directionally consistent with the high PAFs observed in our analysis. Second, the effect of high BMI on ischemic stroke is likely amplified where obesity coexists with poor control of hypertension, diabetes, dyslipidemia, and physical inactivity. Third, limited access to primary prevention, acute stroke care, rehabilitation, and long-term secondary prevention in lower-resource settings may increase both case fatality and post-stroke disability. Together, these mechanisms may help explain why some low- and low-middle SDI settings showed stagnant or worsening HB-IS trends despite overall global declines.

These period-specific PRR patterns add an important equity interpretation to these trends: while successive periods were associated with declining HB-IS DALY rates ratios globally, the direction and magnitude differed by development level. The clearest reductions occurred in high-SDI settings, whereas low, low-middle, and middle SDI settings showed upward shifts over time. This divergence implies that progress in stroke prevention and care has been uneven, potentially widening absolute and relative inequalities among older adults. Strengthening primary care systems for cardiometabolic risk management, ensuring affordable access to essential medicines (23, 29), and improving timely stroke recognition and acute management may be particularly relevant in low-resource contexts, where higher BMI-related HB-IS burden is observed alongside important gaps in prevention and treatment.

Within the ≥70-year population, age patterns were heterogeneous and informative for service planning. Globally, DALY rates increased with advancing age, and sex differences became more apparent in older age bands. High-SDI regions displayed the steepest age-related increases, especially in the oldest age groups, consistent with longer survival into advanced ages and greater accumulation of disability burden. By contrast, flatter or declining patterns at the oldest ages in low and middle SDI regions may reflect competing risks and selective survival, as well as under-ascertainment at very old ages where diagnostic access is limited. These age-pattern differences suggest that high-SDI settings may face a particularly heavy future rehabilitation and long-term care demand among the oldest old, whereas low-SDI settings may experience a growing burden among younger elderly groups that is not fully captured in observed data due to surveillance limitations.

Sex disparities were evident across multiple analyses. Period trends were generally less favorable in males than in females across most SDI strata, and projections suggest a steeper relative increase in male deaths despite females contributing more deaths overall. This pattern indicates that temporal worsening and absolute burden are not necessarily concentrated in the same sex. It may partly reflect sex differences in the accumulation and control of cardiometabolic risk factors across the life course: older men may have greater lifetime exposure to clustered risks such as hypertension, diabetes, dyslipidemia, and smoking, while less consistent engagement with preventive care in some settings may further worsen risk-factor control (26, 30). In addition, differences in body fat distribution and metabolic vulnerability may modify the vascular consequences of high BMI (31), and, in older adults, BMI may incompletely capture central adiposity or sarcopenic obesity (32), which could obscure sex-specific risk patterns. These findings support sex-sensitive rather than one-size-fits-all prevention strategies: intensified screening and management of obesity-related vascular risks may be particularly important for older men with multiple cardiometabolic abnormalities, whereas older women should not be overlooked because they continue to account for a larger share of deaths in absolute terms.

Our projections from 2021 to 2044 highlight a classic “rates down, numbers up” pattern. Although age-specific death rates are projected to continue declining across all older age bands, the absolute number of deaths is expected to increase substantially, with growth increasingly concentrated in the oldest age groups (≥80 years), particularly 80–84, 85–89, and 90–94 years. Total deaths are projected to rise from approximately 80.8 thousand in 2021, based on the model-fitted baseline, to 104.6 thousand in 2044, even as overall death rates decline (both sexes combined). This shift reflects the growing size of the ≥70 population and the increasing share of people surviving into very old age. From a public health perspective, demographic change may sustain a rising absolute HB-IS burden even if current declines in rates continue. Within the GBD attribution framework, these projections support continued attention to life-course prevention and management of obesity and related cardiometabolic risks, together with ongoing improvements in stroke prevention and care, to help moderate future burden in aging populations.

The three-factor decomposition provides a mechanistic demographic explanation for these observed and projected changes. From 1990 to 2021, the net increase in HB-IS deaths was primarily driven by population growth, with population aging contributing modestly, while improvements in age-specific rates offset a large share of the demographic-driven increase. From 2021 to 2044, population growth remains the dominant driver, but the contribution of aging increases markedly, and further rate declines—although substantial—are projected to be insufficient to fully counterbalance the combined demographic pressures. These decomposition results indicate that demographic change is likely to sustain growth in the absolute HB-IS burden, even if rate declines continue. They also support continued attention to prevention, chronic disease management, and health-system preparedness for population aging as part of broader strategies to address future burden among older adults.

A strength of this study is the combination of descriptive mapping, SDI-stratified trend analyses, age/period characterization, decomposition of drivers, and validated forecasting (33). Hindcasting results showed that the primary log-linear model reproduced observed declines with low prediction errors and full coverage within 95% prediction intervals at the global level (34); additional robustness checks indicated broadly consistent declining rate trajectories across alternative model specifications (UCM and ARIMA), supporting the stability of the main projection conclusions. These validation steps increase confidence that the overall direction of projected trends—declining rates but rising counts—reflects structural demographic forces rather than model artifacts.

Several limitations should be considered when interpreting the findings. First, the GBD estimates are model-based and depend on data availability and quality; underreporting, misclassification, and limited diagnostic capacity—especially in low-SDI settings and at the oldest ages—may bias level estimates and obscure true age patterns (35). Second, the GBD comparative risk assessment framework estimates burden attributable to high BMI under standardized assumptions, including the theoretical minimum risk exposure level, and does not fully separate mediation or confounding by related factors such as hypertension, diabetes, diet, and physical inactivity. Therefore, the HB-IS estimates in this study should be interpreted as model-based attributable burden rather than evidence of a direct causal effect of BMI itself. Third, BMI may be a less precise proxy for adiposity in older adults because age-related changes in body composition, including loss of lean mass and sarcopenic obesity, can weaken the correspondence between BMI and metabolic risk. In addition, reverse causation, such as weight loss related to chronic disease or frailty, may influence BMI in advanced age. These considerations may affect attribution and support cautious interpretation of BMI-related HB-IS patterns in this age group. Fourth, the forecasting analysis used empirical age-specific log-linear extrapolation of recent rate and population trends rather than a formal age–period–cohort or Bayesian framework. Accordingly, it assumes that patterns observed over 2000–2021 will continue over the projection horizon and does not explicitly incorporate future structural changes such as obesity pharmacotherapy, shifts in food environments, or major health-system reforms. Uncertainty was assessed through hindcasting, model-based 95% prediction-interval coverage, and alternative-model comparison, and projections should therefore be interpreted as scenario-based estimates under continuation of recent trends rather than precise deterministic forecasts. Fifth, the GBD framework used here does not directly capture longitudinal BMI trajectories, treatment coverage, or health-system access. Therefore, the mechanisms proposed for sex and regional disparities should be interpreted as literature-informed explanations rather than directly tested causal pathways.

Collectively, HB-IS among adults aged ≥70 years shows substantial geographic and socioeconomic heterogeneity, with encouraging declines in rates in high-SDI settings but persistent increases or stagnation in lower-SDI regions. Despite falling rates, absolute deaths are projected to rise through 2044, driven mainly by population growth and accelerated aging, and increasingly concentrated in the oldest age groups. These findings support SDI-tailored actions: strengthening surveillance and essential prevention/treatment capacity in lower-resource settings, while expanding integrated cardiometabolic management, acute stroke systems, rehabilitation, and long-term care planning in higher-SDI settings (36). Within the GBD attribution framework, these findings support continued attention to age- and sex-sensitive obesity prevention and broader metabolic risk management, tailored to local contexts and implemented across the life course, as part of efforts to address future HB-IS burden and related inequalities.

## 5 Conclusion

This study examined the global burden of HB-IS among adults aged 70 years and older from 1990 to 2021, with projections extending to 2044. We found marked geographic, SDI-, sex-, and age-related disparities, with rates declining in higher-SDI settings but remaining stagnant or increasing in lower-SDI regions. Although HB-IS rates are projected to continue decreasing, the absolute number of deaths is expected to rise because of population growth and accelerated aging. These findings underscore the need for SDI-tailored strategies, including enhanced surveillance, obesity prevention and cardiometabolic risk control in older adults, and equitable access to effective stroke prevention and care. The observed disparities may partly reflect heterogeneity in obesity trajectories, cardiometabolic risk-factor control, healthcare access, and socioeconomic context, supporting the need for sex- and region-sensitive prevention strategies.

## Data Availability

The original contributions presented in the study are included in the article/Supplementary material, further inquiries can be directed to the corresponding author.

## Supplementary material

Supplementary Figure S1 Age-specific annual percent change (APC) in HB-IS DALY rates among adults aged ≥70 years by SDI level and sex, 1990–2021.

Supplementary Figure S2 Primary versus sensitivity decomposition of projected HB-IS deaths, 2021–2044.

Supplementary Table S1 Comparison of the most directly relevant recent GBD-based study on high BMI–attributable ischemic stroke and the present study.

Supplementary Table S2 Comparison of 2021–2044 decomposition results using the model-fitted versus descriptive GBD 2021 baseline.

Supplementary Table S3 Observed GBD 2021 versus model-fitted 2021 baseline estimates by age group and sex.

Supplementary Table S4 Breakpoint years, segment intervals, segment-specific APCs, p values, and overall AAPCs for the segmented trend analysis.

Supplementary Table S5 Age-specific APCs in DALY rates by SDI level, sex, and age group.

Supplementary Table S6 PRRs by 5-year period, SDI level, and sex.

Supplementary Table S7 Hindcast performance metrics by outcome and SDI level, including MAPE, MAE, RMSE, and 95% prediction-interval coverage.

Supplementary Table S8 Summary of multi-model robustness comparisons for the global aggregated ≥70-year death-rate series.

## References

[1] Mukherjee D Patil CG. Epidemiology and the global burden of stroke. World Neurosurg. (2011) 76:S85–90. doi: 10.1016/j.wneu.2011.07.023 22182277

[2] Donato AJ Machin DR Lesniewski LA. Mechanisms of dysfunction in the aging vasculature and role in age-related disease. Circ Res. (2018) 123:825–48. doi: 10.1161/CIRCRESAHA.118.312563 30355078 PMC6207260

[3] Pacinella G Ciaccio AM Tuttolomondo A. Endothelial dysfunction and chronic inflammation: the cornerstones of vascular alterations in age-related diseases. Int J Mol Sci. (2022) 23:15722. doi: 10.3390/ijms232415722 36555364 PMC9779461

[4] Guo X Zhang Z Yin X QirongXu Li F Zhu F. Global burden of ischemic stroke attributable to high body mass index in 204 countries and territories, 1990–2021. BMC Cardiovasc Disord. (2024) 24:584. doi: 10.1186/s12872-024-04259-2 39438799 PMC11494805

[5] GBD 2021 Stroke Risk Factor Collaborators. Global, regional, and national burden of stroke and its risk factors, 1990-2021: a systematic analysis for the Global Burden of Disease Study 2021. Lancet Neurol. (2024) 23:973–1003. doi: 10.1016/S1474-4422(24)00369-7 39304265 PMC12254192

[6] Chen X Lu L Xiao C Lan Y Zhong S Qin C . Global burden of ischemic stroke in adults aged 60 years and older from 1990 to 2021: population-based study. PLoS ONE. (2025) 20:e0322606. doi: 10.1371/journal.pone.0322606 40323959 PMC12052125

[7] Stenberg K Hanssen O Edejer TT Bertram M Brindley C Meshreky A . Financing transformative health systems towards achievement of the health sustainable development goals: a model for projected resource needs in 67 low-income and middle-income countries. Lancet Glob Health. (2017) 5:e875–87. doi: 10.1016/S2214-109X(17)30263-2 28728918 PMC5554796

[8] Duncan PW Bushnell C Sissine M Coleman S Lutz BJ Johnson AM . Comprehensive stroke care and outcomes: time for a paradigm shift. Stroke. (2021) 52:385–93. doi: 10.1161/STROKEAHA.120.029678 33349012

[9] Pandian JD Kalkonde Y Sebastian IA Felix C Urimubenshi G Bosch J. Stroke systems of care in low-income and middle-income countries: challenges and opportunities. Lancet. (2020) 396:1443–51. doi: 10.1016/S0140-6736(20)31374-X 33129395

[10] Peer N Baatiema L Kengne AP. Ischaemic heart disease, stroke, and their cardiometabolic risk factors in Africa: current challenges and outlook for the future. Expert Rev Cardiovasc Ther. (2021) 19:129–40. doi: 10.1080/14779072.2021.1855975 33305637

[11] Prust ML Forman R Ovbiagele B. Addressing disparities in the global epidemiology of stroke. Nat Rev Neurol. (2024) 20:207–21. doi: 10.1038/s41582-023-00921-z 38228908

[12] Boot E Ekker MS Putaala J Kittner S De Leeuw FE Tuladhar AM . Ischaemic stroke in young adults: a global perspective. J Neurol Neurosurg Psychiatry. (2020) 91:411–17. doi: 10.1136/jnnp-2019-322424 32015089

[13] Welsh A Hammad M Pina IL Kulinski J. Obesity and cardiovascular health. Eur J Prev Cardiol. (2024) 31:1026–35. doi: 10.1093/eurjpc/zwae025 38243826 PMC11144464

[14] GBD 2019 Diseases and Injuries Collaborators. Global burden of 369 diseases and injuries in 204 countries and territories, 1990–2019: a systematic analysis for the Global Burden of Disease Study 2019. Lancet. (2020) 396:1204–22. doi: 10.1016/S0140-6736(20)30925-9 33069326 PMC7567026

[15] GBD 2021 Causes of Death Collaborators. Global burden of 288 causes of death and life expectancy decomposition in 204 countries and territories and 811 subnational locations, 1990–2021: a systematic analysis for the global burden of disease study 2021. Lancet. (2024) 403:2100–32. doi: 10.1016/S0140-6736(24)00367-2 38582094 PMC11126520

[16] GBD 2021 Risk Factors Collaborators. Global burden and strength of evidence for 88 risk factors in 204 countries and 811 subnational locations, 1990–2021: a systematic analysis for the global burden of disease study 2021. Lancet. (2024) 403:2162–203. doi: 10.1016/S0140-6736(24)00933-4. 38762324 PMC11120204

[17] GBD 2021 Diseases and Injuries Collaborators. Global incidence, prevalence, years lived with disability. (YLDs), disability-adjusted life-years. (DALYs), and healthy life expectancy. (HALE) for 371 diseases and injuries in 204 countries and territories and 811 subnational locations, 1990–2021: a systematic analysis for the global burden of disease study 2021. Lancet. (2024) 403:2133–61. doi: 10.1016/S0140-6736(24)00757-8 38642570 PMC11122111

[18] Stevens GA Alkema L Black RE Boerma JT Collins GS Ezzati M . Guidelines for Accurate and Transparent Health Estimates Reporting: the GATHER statement. Lancet. (2016) 388:e19–23. doi: 10.1016/S0140-6736(16)30388-9 27371184

[19] Feigin VL Roth GA Naghavi M Parmar P Krishnamurthi R Chugh S . Global Burden of Diseases, S. Risk Factors, and G. Stroke Experts Writing, Global burden of stroke and risk factors in 188 countries, during 1990–2013: a systematic analysis for the global burden of disease study 2013. Lancet Neurol. (2016) 15:913–24. doi: 10.1016/S1474-4422(16)30073-4 27291521

[20] Yang S Deng M Ren X Wang F Kong Z Luo J . Global burden of disease study highlights the global, regional and national trends of stroke. J Neurol Neurosurg Psychiatry. (2025) 97:13–24. doi: 10.1136/jnnp-2025-335954 41047223

[21] Flegal KM Panagiotou OA Graubard BI. Estimating population attributable fractions to quantify the health burden of obesity. Ann Epidemiol. (2015) 25:201–7. doi: 10.1016/j.annepidem.2014.11.010 25511307 PMC4383168

[22] Luo Z Shan S Cao J Zhou J Zhou L Jiang D . Temporal trends in cross-country inequalities of stroke and subtypes burden from 1990 to 2021: a secondary analysis of the global burden of disease study 2021. EClinicalMedicine. (2024) 76:102829. doi: 10.1016/j.eclinm.2024.102829 39309727 PMC11415963

[23] Silva GS Rocha E. Developing systems of care for stroke in resource-limited settings. Semin Neurol. (2024) 44:119–29. doi: 10.1055/s-0044-1782617 38513704

[24] O'Brien EC Wu J Zhao X Schulte PJ Fonarow GC Hernandez AF . Healthcare resource availability, quality of care, and acute ischemic stroke outcomes. J Am Heart Assoc. (2017) 6:e003813 doi: 10.1161/JAHA.116.003813 PMC5523738 28159820

[25] Zachrison KS Asif KS Chapman S Joynt Maddox KE Leira EC Maynard S . American Heart association emergency neurovascular, C Telestroke committee of the stroke, c Council on, N Stroke, R council on cardiovascular, and intervention, identifying best practices for improving the evaluation and management of stroke in rural lower-resourced settings: a scientific statement from the American heart association. Stroke. (2025) 56:e62–74. doi: 10.1161/STR.0000000000000478 39665145 PMC12997379

[26] Gu HQ Wang CJ Yang X Liu C Wang X Zhao XQ . Sex differences in vascular risk factors, in-hospital management, and outcomes of patients with acute ischemic stroke in China. Eur J Neurol. (2022) 29:188–98. doi: 10.1111/ene.15124 34564908

[27] Owolabi MO Thrift AG Mahal A Ishida M Martins S Johnson WD . Experts Collaboration, Primary stroke prevention worldwide: translating evidence into action. Lancet Public Health. (2022) 7:e74–85. doi: 10.1016/S2468-2667(21)00281-4 34756176 PMC8727355

[28] NCD Risk Factor Collaboration (NCD-RisC). Worldwide trends in underweight and obesity from 1990 to 2022: a pooled analysis of 3663 population-representative studies with 222 million children, adolescents, and adults. Lancet. (2024) 403:1027–50. doi: 10.1016/S0140-6736(23)02750-2 38432237 PMC7615769

[29] Silva GS Miranda-Alves M. Challenges in the management of stroke in intensive care units in low- and middle-income countries. Neurocrit Care. (2025) 43:1069–77. doi: 10.1007/s12028-025-02319-9 40730698

[30] Hanna M Wabnitz A Grewal P. Sex and stroke risk factors: a review of differences and impact. J Stroke Cerebrovasc Dis. (2024) 33:107624. doi: 10.1016/j.jstrokecerebrovasdis.2024.107624 38316283

[31] Li H Konja D Wang L Wang Y. Sex differences in adiposity and cardiovascular diseases. Int J Mol Sci. (2022) 23:9338 doi: 10.3390/ijms23169338 PMC9409326 36012601

[32] Tan YH Lim JP Lim WS Gao F Teo LLY Ewe SH Keng BMH . Obesity in older adults and associations with cardiovascular structure and function. Obes Facts. (2022) 15:336–43. doi: 10.1159/000521729 35249039 PMC9209947

[33] Gao N Yang N Huang J. Decomposition, forecasting of colorectal cancer burden attributable to high body mass index, high fasting plasma glucose, et al. Front Nutr. (2025) 12:1652676. doi: 10.3389/fnut.2025.1652676 41479670 PMC12753452

[34] Rydevik G Innocent GT Marion G Davidson RS White PC Billinis C . Hutchings, using combined diagnostic test results to hindcast trends of infection from cross-sectional data. PLoS Comput Biol. (2016) 12:e1004901. doi: 10.1371/journal.pcbi.1004901 27384712 PMC4934910

[35] Liu C Shi T Li S Wu S Chen J Cai C . Global, regional, and national liver cancer attributable to smoking and alcohol use burden, 1990–2021: analysis for the global burden of disease 2021 study. BMC Public Health. (2025) 25:2037. doi: 10.1186/s12889-025-23184-3 40457284 PMC12128314

[36] Feigin VL Owolabi MO. G World stroke organization–lancet neurology commission stroke collaboration group. Pragmatic solutions to reduce the global burden of stroke: a world stroke organization-lancet neurology commission. Lancet Neurol. (2023) 22:1160–06. doi: 10.1016/S1474-4422(23)00277-6 37827183 PMC10715732

