# Supplementary figures and images for: Global, regional, and national burden of disease for high BMI-related ischemic stroke in people aged 70 and older: trend analysis from 1990 to 2021 and projections for 2044
Source: Front Neurol. 2026 May 22;17:1794665. doi: 10.3389/fneur.2026.1794665 (PMC13236533; doi:10.3389/fneur.2026.1794665)

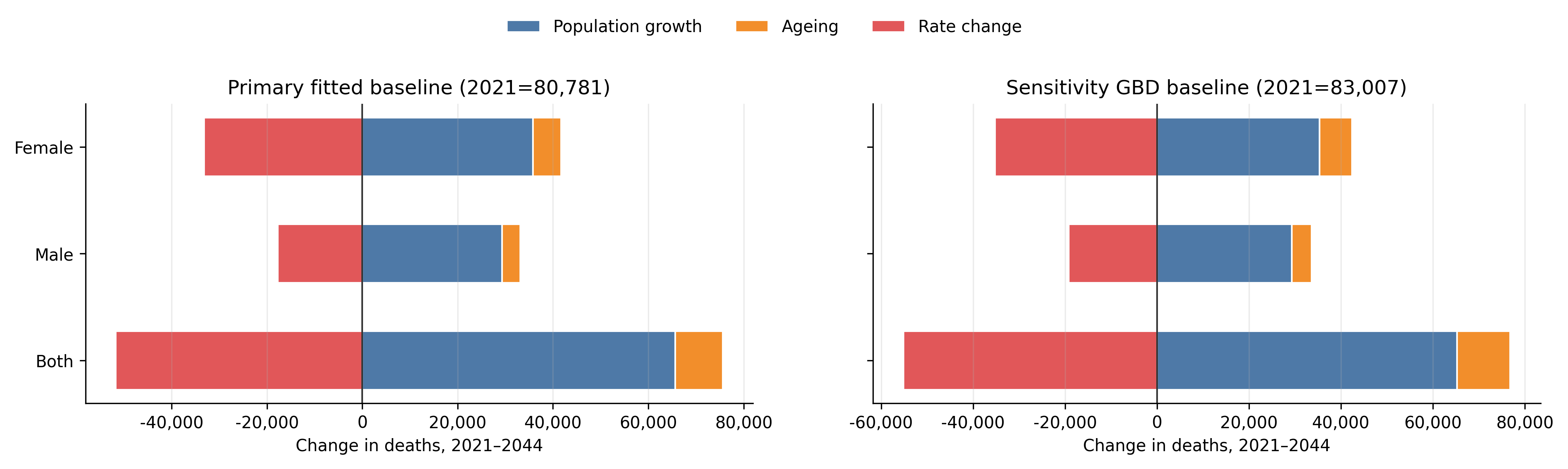

Supplement: Supplementary Figure S1 — Age-specific annual percent change (APC) in HB-IS DALY rates among adults aged ≥70 years by SDI level and sex, 1990–2021. [file Image_1.png]

Annual change (% per year)

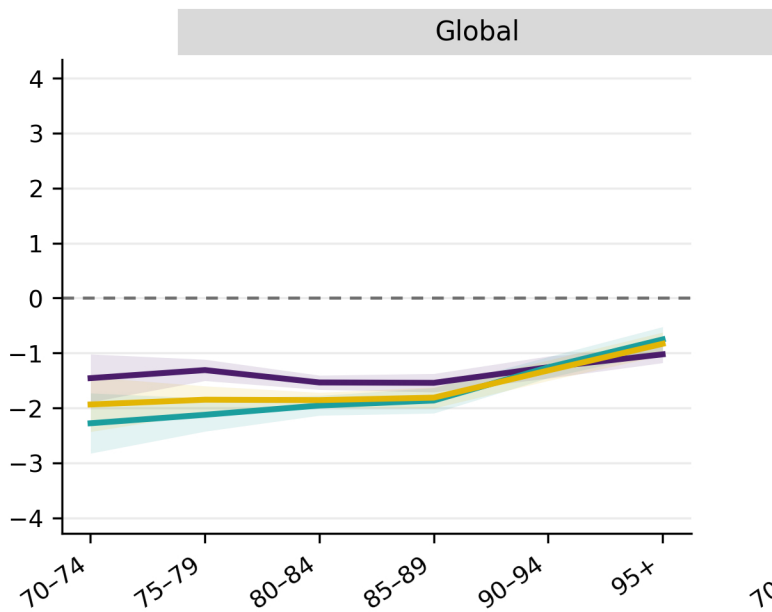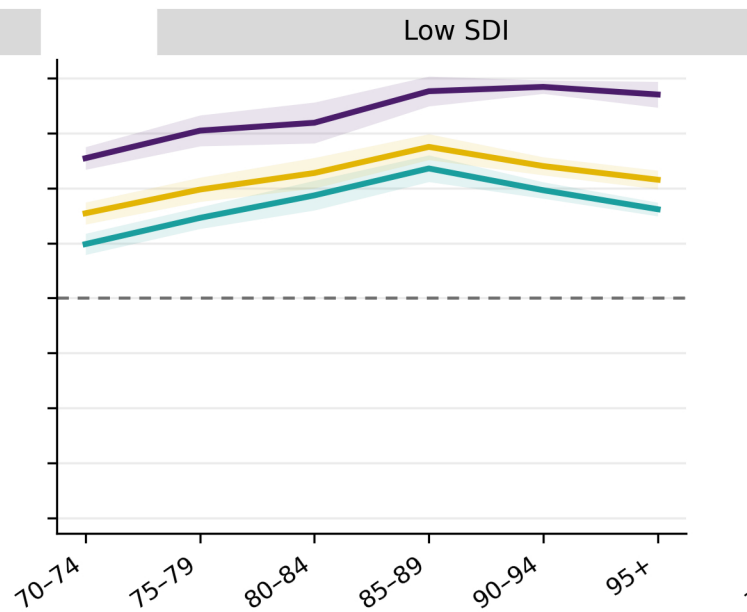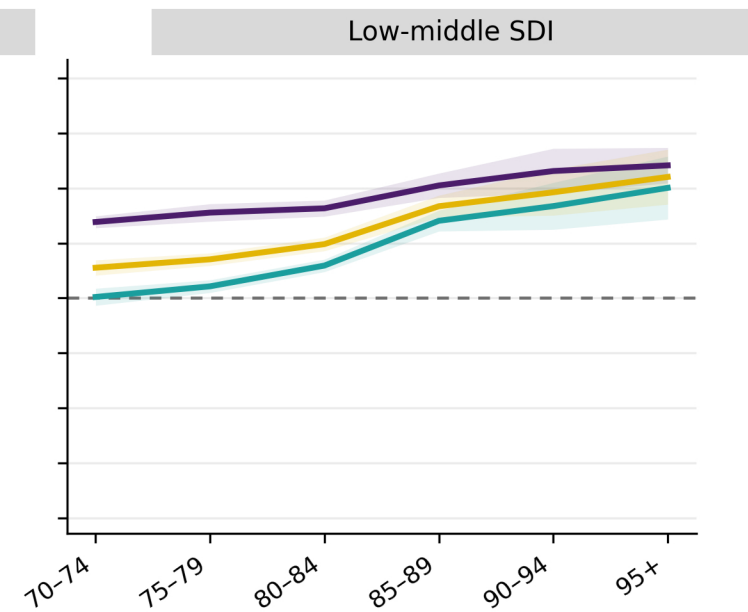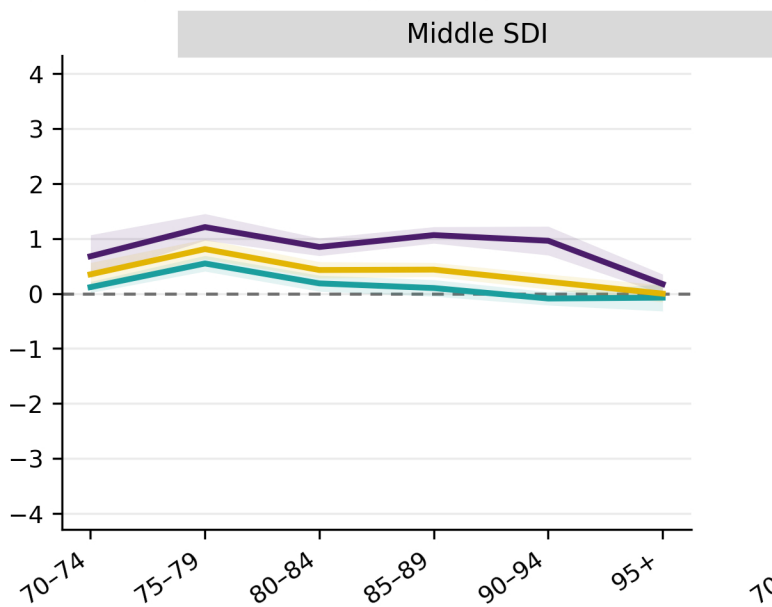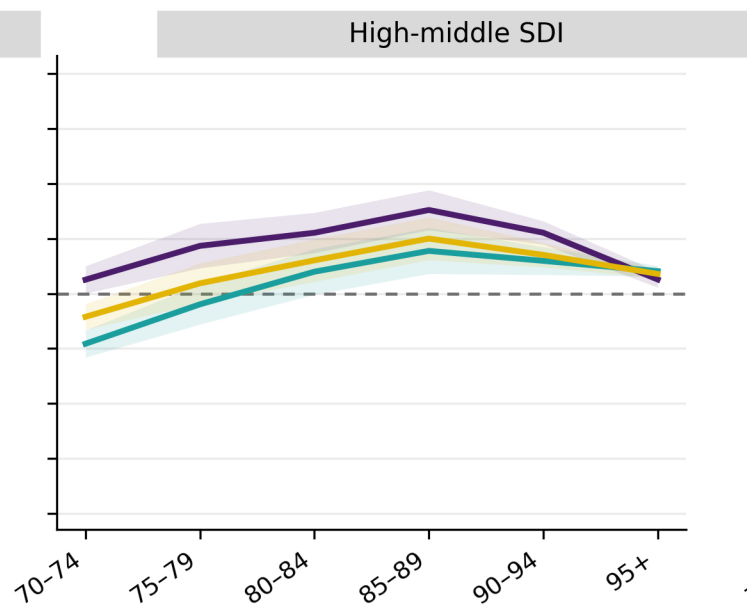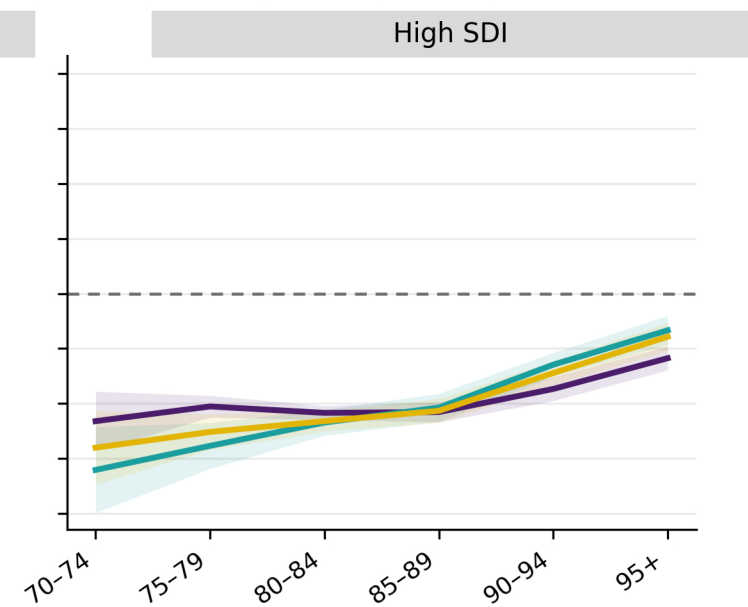

group

- Male
- Female
- Both

Supplement: Supplementary Figure S2 — Primary versus sensitivity decomposition of projected HB-IS deaths, 2021–2044. [file Data_Sheet_1.pdf]
