# Supplementary material for: Global, regional, and national burden of disease for high BMI-related ischemic stroke in people aged 70 and older: trend analysis from 1990 to 2021 and projections for 2044
Source: Front Neurol. 2026 May 22;17:1794665. doi: 10.3389/fneur.2026.1794665 (PMC13236533; doi:10.3389/fneur.2026.1794665)
Supplement: Supplementary Table S1 — Comparison of the most directly relevant recent GBD-based study on high BMI–attributable ischemic stroke and the present study. [file Table_1.docx]

**Supplementary Table S1.** Comparison of the most directly relevant recent GBD-based study on high BMI–attributable ischemic stroke and the present study

| **Study** | **Population** | **Age handling** | **Outcomes** | **Trend metrics** | **Within-elderly analysis** | **Forecasting** | **Decomposition / policy implication** |
| --- | --- | --- | --- | --- | --- | --- | --- |
| Guo et al., 2024 | Global, regional, and national burden of ischemic stroke attributable to high BMI in 204 countries and territories, 1990–2021; all ages | Age and sex stratified descriptive analyses across the full age spectrum (16 age groups); older age groups were described, but adults aged ≥70 years were not treated as the analytic population itself | Deaths, DALYs, ASMR, ASDR | EAPC for ASMR/ASDR; associations with SDI, HDI, and baseline ASR | The study reported descriptive age patterns, but did not use an elderly restricted framework with 5-year elderly age bands, PRR-based period effects, or elderly-specific segmented trend modelling | Not performed | No formal decomposition; policy implications emphasized country specific and SDI aware prevention and control strategies |
| Present study | Global, regional, and national HB-IS burden in adults aged ≥70 years only, 1990–2021, with projections to 2044 | Elderly-restricted analytic framework with 5-year age bands: 70–74, 75–79, 80–84, 85–89, 90–94, and ≥95 years | Deaths, DALYs, death/DALY rates, and PAFs | Segmented log-linear regression with APC/AAPC; PRRs for period effects; age-specific APC analyses | Explicit characterization of within elderly heterogeneity, including age gradients within ≥70 years and divergence by SDI and sex | 2022–2044 projections with hindcasting validation and UCM/ARIMA robustness checks | Shapley based three factor decomposition quantified the contributions of population growth, population ageing, and rate change, supporting ageing focused and SDI-tailored prevention and care planning |

HB-IS, high BMI–attributable ischemic stroke; DALYs, disability-adjusted life years; ASMR, age-standardized mortality rate; ASDR, age-standardized disability-adjusted life year rate; EAPC, estimated annual percentage change; APC, annual percent change; AAPC, average annual percent change; PRR, period rate ratio; PAF, population-attributable fraction.

**Supplementary Table S2.** Comparison of 2021–2044 decomposition results using the model-fitted versus descriptive GBD 2021 baseline

| **Sex** | **2021 baseline deaths (primary fitted)** | **2044 deaths (primary fitted)** | **Net change, 2021–2044 (primary fitted)** | **Population growth (primary fitted)** | **Population ageing (primary fitted)** | **Rate change (primary fitted)** | **2021 baseline deaths (GBD baseline)** | **2044 deaths (GBD baseline)** | **Net change, 2021–2044 (GBD baseline)** | **Population growth (GBD baseline)** | **Population ageing (GBD baseline)** | **Rate change (GBD baseline)** | **Difference in 2021 baseline deaths (abs)** | **Difference in 2021 baseline deaths (% vs primary)** | **Difference in net change (abs)** | **Difference in net change (% vs primary)** | **Difference in population growth (abs)** | **Difference in population growth (% vs primary)** | **Difference in population ageing (abs)** | **Difference in population ageing (% vs primary)** | **Difference in rate change (abs)** | **Difference in rate change (% vs primary)** |
| --- | --- | --- | --- | --- | --- | --- | --- | --- | --- | --- | --- | --- | --- | --- | --- | --- | --- | --- | --- | --- | --- | --- |
| Both | 80,780.84 | 104,638.41 | 23,857.58 | 65,657.88 | 9,927.40 | -51,727.70 | 83,006.65 | 104,638.41 | 21,631.76 | 65,218.40 | 11,578.90 | -55,165.55 | 2,225.81 | 2.76 | -2,225.81 | -9.33 | -439.47 | -0.67 | 1,651.50 | 16.64 | -3,437.85 | 6.65 |
| Male | 31,260.97 | 46,612.56 | 15,351.59 | 29,314.00 | 3,818.61 | -17,781.02 | 32,189.59 | 46,612.56 | 14,422.97 | 29,273.04 | 4,376.58 | -19,226.65 | 928.62 | 2.97 | -928.62 | -6.05 | -40.96 | -0.14 | 557.97 | 14.61 | -1,445.63 | 8.13 |
| Female | 49,519.86 | 58,025.85 | 8,505.99 | 35,766.76 | 5,960.46 | -33,221.24 | 50,817.06 | 58,025.85 | 7,208.79 | 35,362.24 | 7,065.32 | -35,218.77 | 1,297.20 | 2.62 | -1,297.20 | -15.25 | -404.52 | -1.13 | 1,104.86 | 18.54 | -1,997.53 | 6.01 |

**Supplementary Table S3.** Observed GBD 2021 versus model-fitted 2021 baseline estimates by age group and sex

| **Sex** | **Age group** | **Observed deaths (2021)** | **Observed rate (per 100,000)** | **Observed population (2021)** | **Fitted deaths (2021)** | **Fitted rate (per 100,000)** | **Fitted population (2021)** | **Difference in deaths (abs)** | **Difference in deaths (% vs fitted)** | **Difference in rate (abs)** | **Difference in rate (% vs fitted)** | **Difference in population (abs)** | **Difference in population (% vs fitted)** |
| --- | --- | --- | --- | --- | --- | --- | --- | --- | --- | --- | --- | --- | --- |
| Both | 70-74 years | 22,798.15 | 11.04 | 206,504,981.90 | 18,992.71 | 10.01 | 189,815,972.40 | 3,805.44 | 20.04 | 1.03 | 10.34 | 16,689,009.51 | 8.79 |
| Both | 75-79 years | 18,223.09 | 13.77 | 132,339,070.40 | 18,923.06 | 13.92 | 135,985,322.80 | -699.97 | -3.70 | -0.15 | -1.05 | -3,646,252.32 | -2.68 |
| Both | 80-84 years | 17,411.22 | 19.41 | 89,702,318.39 | 18,068.87 | 19.30 | 93,618,562.60 | -657.65 | -3.64 | 0.11 | 0.57 | -3,916,244.20 | -4.18 |
| Both | 85-89 years | 14,048.94 | 29.16 | 48,178,806.58 | 14,811.04 | 29.54 | 50,144,460.28 | -762.10 | -5.15 | -0.38 | -1.28 | -1,965,653.70 | -3.92 |
| Both | 90-94 years | 7,838.06 | 41.86 | 18,724,462.49 | 7,439.62 | 39.68 | 18,746,848.12 | 398.44 | 5.36 | 2.18 | 5.48 | -22,385.62 | -0.12 |
| Both | 95+ years | 2,687.19 | 47.78 | 5,624,089.58 | 2,545.54 | 45.19 | 5,632,807.93 | 141.65 | 5.56 | 2.59 | 5.73 | -8,718.36 | -0.15 |
| Male | 70-74 years | 10,794.70 | 11.15 | 96,813,452.91 | 9,030.25 | 10.10 | 89,442,747.63 | 1,764.45 | 19.54 | 1.05 | 10.44 | 7,370,705.29 | 8.24 |
| Male | 75-79 years | 7,688.47 | 12.82 | 59,972,464.90 | 8,068.48 | 13.07 | 61,750,861.63 | -380.01 | -4.71 | -0.25 | -1.88 | -1,778,396.73 | -2.88 |
| Male | 80-84 years | 6,346.08 | 16.81 | 37,751,814.40 | 6,763.09 | 16.94 | 39,924,624.07 | -417.01 | -6.17 | -0.13 | -0.77 | -2,172,809.68 | -5.44 |
| Male | 85-89 years | 4,779.64 | 25.69 | 18,605,060.33 | 4,988.97 | 25.62 | 19,470,942.84 | -209.33 | -4.20 | 0.07 | 0.26 | -865,882.51 | -4.45 |
| Male | 90-94 years | 2,010.38 | 31.59 | 6,363,975.94 | 1,867.34 | 29.56 | 6,317,847.34 | 143.04 | 7.66 | 2.03 | 6.88 | 46,128.60 | 0.73 |
| Male | 95+ years | 570.32 | 35.71 | 1,597,087.65 | 542.85 | 34.46 | 1,575,325.77 | 27.47 | 5.06 | 1.25 | 3.63 | 21,761.88 | 1.38 |
| Female | 70-74 years | 12,003.45 | 10.94 | 109,720,749.50 | 9,962.47 | 9.93 | 100,373,224.70 | 2,040.98 | 20.49 | 1.01 | 10.22 | 9,347,524.80 | 9.31 |
| Female | 75-79 years | 10,534.62 | 14.57 | 72,303,500.34 | 10,854.58 | 14.62 | 74,234,461.13 | -319.96 | -2.95 | -0.05 | -0.36 | -1,930,960.79 | -2.60 |
| Female | 80-84 years | 11,065.14 | 21.31 | 51,924,636.32 | 11,305.78 | 21.06 | 53,693,938.52 | -240.64 | -2.13 | 0.25 | 1.21 | -1,769,302.20 | -3.30 |
| Female | 85-89 years | 9,269.30 | 31.34 | 29,576,579.45 | 9,822.07 | 32.02 | 30,673,517.43 | -552.77 | -5.63 | -0.68 | -2.13 | -1,096,937.98 | -3.58 |
| Female | 90-94 years | 5,827.68 | 47.15 | 12,359,872.75 | 5,572.28 | 44.83 | 12,429,000.78 | 255.40 | 4.58 | 2.32 | 5.17 | -69,128.03 | -0.56 |
| Female | 95+ years | 2,116.87 | 52.56 | 4,027,530.44 | 2,002.69 | 49.36 | 4,057,482.16 | 114.18 | 5.70 | 3.20 | 6.49 | -29,951.72 | -0.74 |
| Both | All 70+ ages | 83,006.65 | 16.57 | 501,073,729.40 | 80,780.84 | 16.35 | 493,943,974.10 | 2,225.81 | 2.76 | 0.21 | 1.29 | 7,129,755.32 | 1.44 |
| Male | All 70+ ages | 32,189.59 | 14.56 | 221,103,856.10 | 31,260.97 | 14.31 | 218,482,349.30 | 928.62 | 2.97 | 0.25 | 1.75 | 2,621,506.85 | 1.20 |
| Female | All 70+ ages | 50,817.06 | 18.15 | 279,912,868.80 | 49,519.86 | 17.98 | 275,461,624.80 | 1,297.20 | 2.62 | 0.18 | 0.99 | 4,451,244.07 | 1.62 |

**Supplementary Table S4.** Breakpoint years, segment intervals, segment-specific APCs, p values, and overall AAPCs for the segmented trend analysis

| **Measure** | **Sex** | **Location** | **No. of segments** | **Breakpoint year(s)** | **Segment no.** | **Segment interval** | **APC (%)** | **APC p value** | **APC significance** | **AAPC period** | **AAPC (%)** | **AAPC p value** | **AAPC significance** |
| --- | --- | --- | --- | --- | --- | --- | --- | --- | --- | --- | --- | --- | --- |
| Deaths | Both | Global | 3 | 2002, 2007 | 1 | 1990–2002 | -0.45 | <0.001 | *** | 1990–2021 | -1.82 | <0.0001 | *** |
| Deaths | Both | Global | 3 | 2002, 2007 | 2 | 2002–2007 | -3.40 | <0.0001 | *** | 1990–2021 | -1.82 | <0.0001 | *** |
| Deaths | Both | Global | 3 | 2002, 2007 | 3 | 2007–2021 | -2.00 | <0.0001 | *** | 1990–2021 | -1.82 | <0.0001 | *** |
| Deaths | Both | Low SDI | 3 | 1998, 2013 | 1 | 1990–1998 | 1.47 | <0.0001 | *** | 1990–2021 | 1.89 | <0.0001 | *** |
| Deaths | Both | Low SDI | 3 | 1998, 2013 | 2 | 1998–2013 | 2.52 | <0.0001 | *** | 1990–2021 | 1.89 | <0.0001 | *** |
| Deaths | Both | Low SDI | 3 | 1998, 2013 | 3 | 2013–2021 | -0.12 | 0.377 | ns | 1990–2021 | 1.89 | <0.0001 | *** |
| Deaths | Both | Low-middle SDI | 2 | 2003 | 1 | 1990–2003 | 0.25 | <0.001 | *** | 1990–2021 | 0.80 | <0.0001 | *** |
| Deaths | Both | Low-middle SDI | 2 | 2003 | 2 | 2003–2021 | 1.15 | <0.0001 | *** | 1990–2021 | 0.80 | <0.0001 | *** |
| Deaths | Both | Middle SDI | 2 | 2005 | 1 | 1990–2005 | 1.01 | <0.0001 | *** | 1990–2021 | 0.46 | <0.0001 | *** |
| Deaths | Both | Middle SDI | 2 | 2005 | 2 | 2005–2021 | -0.02 | 0.701 | ns | 1990–2021 | 0.46 | <0.0001 | *** |
| Deaths | Both | High-middle SDI | 2 | 2016 | 1 | 1990–2016 | 0.39 | <0.0001 | *** | 1990–2021 | 0.07 | 0.680 | ns |
| Deaths | Both | High-middle SDI | 2 | 2016 | 2 | 2016–2021 | -3.74 | <0.0001 | *** | 1990–2021 | 0.07 | 0.680 | ns |
| Deaths | Both | High SDI | 3 | 2002, 2011 | 1 | 1990–2002 | -0.59 | <0.0001 | *** | 1990–2021 | -2.36 | <0.0001 | *** |
| Deaths | Both | High SDI | 3 | 2002, 2011 | 2 | 2002–2011 | -3.73 | <0.0001 | *** | 1990–2021 | -2.36 | <0.0001 | *** |
| Deaths | Both | High SDI | 3 | 2002, 2011 | 3 | 2011–2021 | -2.37 | <0.0001 | *** | 1990–2021 | -2.36 | <0.0001 | *** |
| Deaths | Male | Global | 2 | 1999 | 1 | 1990–1999 | -0.41 | <0.0001 | *** | 1990–2021 | -1.49 | <0.0001 | *** |
| Deaths | Male | Global | 2 | 1999 | 2 | 1999–2021 | -1.79 | <0.0001 | *** | 1990–2021 | -1.49 | <0.0001 | *** |
| Deaths | Male | Low SDI | 3 | 2003, 2012 | 1 | 1990–2003 | 2.97 | <0.0001 | *** | 1990–2021 | 2.87 | <0.0001 | *** |
| Deaths | Male | Low SDI | 3 | 2003, 2012 | 2 | 2003–2012 | 3.86 | <0.0001 | *** | 1990–2021 | 2.87 | <0.0001 | *** |
| Deaths | Male | Low SDI | 3 | 2003, 2012 | 3 | 2012–2021 | 0.62 | 0.003 | ** | 1990–2021 | 2.87 | <0.0001 | *** |
| Deaths | Male | Low-middle SDI | 3 | 2005, 2016 | 1 | 1990–2005 | 1.08 | <0.0001 | *** | 1990–2021 | 1.53 | <0.0001 | *** |
| Deaths | Male | Low-middle SDI | 3 | 2005, 2016 | 2 | 2005–2016 | 2.15 | <0.0001 | *** | 1990–2021 | 1.53 | <0.0001 | *** |
| Deaths | Male | Low-middle SDI | 3 | 2005, 2016 | 3 | 2016–2021 | 0.55 | <0.0001 | *** | 1990–2021 | 1.53 | <0.0001 | *** |
| Deaths | Male | Middle SDI | 2 | 2003 | 1 | 1990–2003 | 1.92 | <0.0001 | *** | 1990–2021 | 0.85 | <0.0001 | *** |
| Deaths | Male | Middle SDI | 2 | 2003 | 2 | 2003–2021 | 0.23 | <0.001 | *** | 1990–2021 | 0.85 | <0.0001 | *** |
| Deaths | Male | High-middle SDI | 2 | 2016 | 1 | 1990–2016 | 0.95 | <0.0001 | *** | 1990–2021 | 0.62 | <0.001 | *** |
| Deaths | Male | High-middle SDI | 2 | 2016 | 2 | 2016–2021 | -3.14 | <0.0001 | *** | 1990–2021 | 0.62 | <0.001 | *** |
| Deaths | Male | High SDI | 3 | 2002, 2012 | 1 | 1990–2002 | -0.97 | <0.0001 | *** | 1990–2021 | -2.25 | <0.0001 | *** |
| Deaths | Male | High SDI | 3 | 2002, 2012 | 2 | 2002–2012 | -3.29 | <0.0001 | *** | 1990–2021 | -2.25 | <0.0001 | *** |
| Deaths | Male | High SDI | 3 | 2002, 2012 | 3 | 2012–2021 | -1.96 | <0.0001 | *** | 1990–2021 | -2.25 | <0.0001 | *** |
| Deaths | Female | Global | 3 | 2002, 2007 | 1 | 1990–2002 | -0.33 | 0.012 | * | 1990–2021 | -1.95 | <0.0001 | *** |
| Deaths | Female | Global | 3 | 2002, 2007 | 2 | 2002–2007 | -3.65 | <0.0001 | *** | 1990–2021 | -1.95 | <0.0001 | *** |
| Deaths | Female | Global | 3 | 2002, 2007 | 3 | 2007–2021 | -2.19 | <0.0001 | *** | 1990–2021 | -1.95 | <0.0001 | *** |
| Deaths | Female | Low SDI | 3 | 1997, 2014 | 1 | 1990–1997 | 0.97 | <0.0001 | *** | 1990–2021 | 1.40 | <0.0001 | *** |
| Deaths | Female | Low SDI | 3 | 1997, 2014 | 2 | 1997–2014 | 1.89 | <0.0001 | *** | 1990–2021 | 1.40 | <0.0001 | *** |
| Deaths | Female | Low SDI | 3 | 1997, 2014 | 3 | 2014–2021 | -0.61 | <0.0001 | *** | 1990–2021 | 1.40 | <0.0001 | *** |
| Deaths | Female | Low-middle SDI | 3 | 2002, 2009 | 1 | 1990–2002 | -0.16 | 0.072 | ns | 1990–2021 | 0.35 | <0.0001 | *** |
| Deaths | Female | Low-middle SDI | 3 | 2002, 2009 | 2 | 2002–2009 | 0.29 | <0.0001 | *** | 1990–2021 | 0.35 | <0.0001 | *** |
| Deaths | Female | Low-middle SDI | 3 | 2002, 2009 | 3 | 2009–2021 | 0.97 | <0.0001 | *** | 1990–2021 | 0.35 | <0.0001 | *** |
| Deaths | Female | Middle SDI | 2 | 2006 | 1 | 1990–2006 | 0.57 | <0.0001 | *** | 1990–2021 | 0.21 | <0.0001 | *** |
| Deaths | Female | Middle SDI | 2 | 2006 | 2 | 2006–2021 | -0.22 | <0.0001 | *** | 1990–2021 | 0.21 | <0.0001 | *** |
| Deaths | Female | High-middle SDI | 2 | 2016 | 1 | 1990–2016 | 0.08 | 0.410 | ns | 1990–2021 | -0.25 | 0.148 | ns |
| Deaths | Female | High-middle SDI | 2 | 2016 | 2 | 2016–2021 | -4.17 | <0.0001 | *** | 1990–2021 | -0.25 | 0.148 | ns |
| Deaths | Female | High SDI | 3 | 2002, 2011 | 1 | 1990–2002 | -0.33 | 0.029 | * | 1990–2021 | -2.36 | <0.0001 | *** |
| Deaths | Female | High SDI | 3 | 2002, 2011 | 2 | 2002–2011 | -3.88 | <0.0001 | *** | 1990–2021 | -2.36 | <0.0001 | *** |
| Deaths | Female | High SDI | 3 | 2002, 2011 | 3 | 2011–2021 | -2.53 | <0.0001 | *** | 1990–2021 | -2.36 | <0.0001 | *** |
| DALYs | Both | Global | 3 | 2001, 2012 | 1 | 1990–2001 | -0.06 | 0.615 | ns | 1990–2021 | -1.81 | <0.0001 | *** |
| DALYs | Both | Global | 3 | 2001, 2012 | 2 | 2001–2012 | -2.85 | <0.0001 | *** | 1990–2021 | -1.81 | <0.0001 | *** |
| DALYs | Both | Global | 3 | 2001, 2012 | 3 | 2012–2021 | -1.71 | <0.0001 | *** | 1990–2021 | -1.81 | <0.0001 | *** |
| DALYs | Both | Low SDI | 3 | 1998, 2013 | 1 | 1990–1998 | 1.38 | <0.0001 | *** | 1990–2021 | 1.79 | <0.0001 | *** |
| DALYs | Both | Low SDI | 3 | 1998, 2013 | 2 | 1998–2013 | 2.37 | <0.0001 | *** | 1990–2021 | 1.79 | <0.0001 | *** |
| DALYs | Both | Low SDI | 3 | 1998, 2013 | 3 | 2013–2021 | -0.08 | 0.551 | ns | 1990–2021 | 1.79 | <0.0001 | *** |
| DALYs | Both | Low-middle SDI | 2 | 2002 | 1 | 1990–2002 | 0.15 | 0.055 | ns | 1990–2021 | 0.72 | <0.0001 | *** |
| DALYs | Both | Low-middle SDI | 2 | 2002 | 2 | 2002–2021 | 1.02 | <0.0001 | *** | 1990–2021 | 0.72 | <0.0001 | *** |
| DALYs | Both | Middle SDI | 2 | 2004 | 1 | 1990–2004 | 1.11 | <0.0001 | *** | 1990–2021 | 0.45 | <0.0001 | *** |
| DALYs | Both | Middle SDI | 2 | 2004 | 2 | 2004–2021 | -0.02 | 0.588 | ns | 1990–2021 | 0.45 | <0.0001 | *** |
| DALYs | Both | High-middle SDI | 2 | 2002 | 1 | 1990–2002 | 1.12 | <0.0001 | *** | 1990–2021 | -0.04 | 0.768 | ns |
| DALYs | Both | High-middle SDI | 2 | 2002 | 2 | 2002–2021 | -0.63 | <0.001 | *** | 1990–2021 | -0.04 | 0.768 | ns |
| DALYs | Both | High SDI | 3 | 2002, 2013 | 1 | 1990–2002 | -0.21 | 0.114 | ns | 1990–2021 | -2.39 | <0.0001 | *** |
| DALYs | Both | High SDI | 3 | 2002, 2013 | 2 | 2002–2013 | -3.84 | <0.0001 | *** | 1990–2021 | -2.39 | <0.0001 | *** |
| DALYs | Both | High SDI | 3 | 2002, 2013 | 3 | 2013–2021 | -2.12 | <0.0001 | *** | 1990–2021 | -2.39 | <0.0001 | *** |
| DALYs | Male | Global | 3 | 2001, 2013 | 1 | 1990–2001 | -0.04 | 0.638 | ns | 1990–2021 | -1.42 | <0.0001 | *** |
| DALYs | Male | Global | 3 | 2001, 2013 | 2 | 2001–2013 | -2.24 | <0.0001 | *** | 1990–2021 | -1.42 | <0.0001 | *** |
| DALYs | Male | Global | 3 | 2001, 2013 | 3 | 2013–2021 | -1.16 | <0.0001 | *** | 1990–2021 | -1.42 | <0.0001 | *** |
| DALYs | Male | Low SDI | 3 | 2003, 2012 | 1 | 1990–2003 | 2.84 | <0.0001 | *** | 1990–2021 | 2.77 | <0.0001 | *** |
| DALYs | Male | Low SDI | 3 | 2003, 2012 | 2 | 2003–2012 | 3.65 | <0.0001 | *** | 1990–2021 | 2.77 | <0.0001 | *** |
| DALYs | Male | Low SDI | 3 | 2003, 2012 | 3 | 2012–2021 | 0.77 | <0.0001 | *** | 1990–2021 | 2.77 | <0.0001 | *** |
| DALYs | Male | Low-middle SDI | 3 | 2004, 2015 | 1 | 1990–2004 | 1.07 | <0.0001 | *** | 1990–2021 | 1.49 | <0.0001 | *** |
| DALYs | Male | Low-middle SDI | 3 | 2004, 2015 | 2 | 2004–2015 | 2.07 | <0.0001 | *** | 1990–2021 | 1.49 | <0.0001 | *** |
| DALYs | Male | Low-middle SDI | 3 | 2004, 2015 | 3 | 2015–2021 | 0.69 | <0.0001 | *** | 1990–2021 | 1.49 | <0.0001 | *** |
| DALYs | Male | Middle SDI | 2 | 2002 | 1 | 1990–2002 | 2.04 | <0.0001 | *** | 1990–2021 | 0.83 | <0.0001 | *** |
| DALYs | Male | Middle SDI | 2 | 2002 | 2 | 2002–2021 | 0.20 | <0.0001 | *** | 1990–2021 | 0.83 | <0.0001 | *** |
| DALYs | Male | High-middle SDI | 2 | 2010 | 1 | 1990–2010 | 1.08 | <0.0001 | *** | 1990–2021 | 0.53 | <0.001 | *** |
| DALYs | Male | High-middle SDI | 2 | 2010 | 2 | 2010–2021 | -0.76 | 0.002 | ** | 1990–2021 | 0.53 | <0.001 | *** |
| DALYs | Male | High SDI | 3 | 2002, 2014 | 1 | 1990–2002 | -0.43 | <0.0001 | *** | 1990–2021 | -2.15 | <0.0001 | *** |
| DALYs | Male | High SDI | 3 | 2002, 2014 | 2 | 2002–2014 | -3.37 | <0.0001 | *** | 1990–2021 | -2.15 | <0.0001 | *** |
| DALYs | Male | High SDI | 3 | 2002, 2014 | 3 | 2014–2021 | -1.45 | <0.0001 | *** | 1990–2021 | -2.15 | <0.0001 | *** |
| DALYs | Female | Global | 3 | 2001, 2012 | 1 | 1990–2001 | -0.02 | 0.908 | ns | 1990–2021 | -2.01 | <0.0001 | *** |
| DALYs | Female | Global | 3 | 2001, 2012 | 2 | 2001–2012 | -3.18 | <0.0001 | *** | 1990–2021 | -2.01 | <0.0001 | *** |
| DALYs | Female | Global | 3 | 2001, 2012 | 3 | 2012–2021 | -2.00 | <0.0001 | *** | 1990–2021 | -2.01 | <0.0001 | *** |
| DALYs | Female | Low SDI | 3 | 1997, 2014 | 1 | 1990–1997 | 0.88 | <0.0001 | *** | 1990–2021 | 1.29 | <0.0001 | *** |
| DALYs | Female | Low SDI | 3 | 1997, 2014 | 2 | 1997–2014 | 1.75 | <0.0001 | *** | 1990–2021 | 1.29 | <0.0001 | *** |
| DALYs | Female | Low SDI | 3 | 1997, 2014 | 3 | 2014–2021 | -0.62 | <0.0001 | *** | 1990–2021 | 1.29 | <0.0001 | *** |
| DALYs | Female | Low-middle SDI | 2 | 2004 | 1 | 1990–2004 | -0.25 | <0.001 | *** | 1990–2021 | 0.24 | <0.001 | *** |
| DALYs | Female | Low-middle SDI | 2 | 2004 | 2 | 2004–2021 | 0.62 | <0.0001 | *** | 1990–2021 | 0.24 | <0.001 | *** |
| DALYs | Female | Middle SDI | 2 | 2005 | 1 | 1990–2005 | 0.59 | <0.0001 | *** | 1990–2021 | 0.19 | <0.001 | *** |
| DALYs | Female | Middle SDI | 2 | 2005 | 2 | 2005–2021 | -0.19 | <0.001 | *** | 1990–2021 | 0.19 | <0.001 | *** |
| DALYs | Female | High-middle SDI | 2 | 2001 | 1 | 1990–2001 | 0.98 | <0.0001 | *** | 1990–2021 | -0.40 | 0.010 | ** |
| DALYs | Female | High-middle SDI | 2 | 2001 | 2 | 2001–2021 | -1.00 | <0.0001 | *** | 1990–2021 | -0.40 | 0.010 | ** |
| DALYs | Female | High SDI | 3 | 2002, 2013 | 1 | 1990–2002 | -0.05 | 0.742 | ns | 1990–2021 | -2.51 | <0.0001 | *** |
| DALYs | Female | High SDI | 3 | 2002, 2013 | 2 | 2002–2013 | -4.09 | <0.0001 | *** | 1990–2021 | -2.51 | <0.0001 | *** |
| DALYs | Female | High SDI | 3 | 2002, 2013 | 3 | 2013–2021 | -2.48 | <0.0001 | *** | 1990–2021 | -2.51 | <0.0001 | *** |

APC, annual percent change; AAPC, average annual percent change. Breakpoint years were selected by BIC from segmented log-linear regression with integer-year joinpoints and a minimum segment length of 5 years. Segment-specific APCs and overall AAPCs were estimated from log-linear models using heteroskedasticity- and autocorrelation-consistent (HAC) standard errors (maxlag = 1). Overall AAPC was estimated from a separate full-period model (1990–2021) within each stratum.*p < 0.05; **p < 0.01; ***p < 0.001; ns, not significant.

**Supplementary Table S5.** Age-specific APCs in DALY rates by SDI level, sex, and age group

| **Measure** | **Location** | **Sex** | **Age group** | **Years** | **APC (95% CI)** | **P (formatted)** | **Sig.** |
| --- | --- | --- | --- | --- | --- | --- | --- |
| DALYs | Global | Male | 70–74 years | 1990–2021 | -1.457 (-1.892 — -1.020) | <0.0001 | *** |
| DALYs | Global | Male | 75–79 years | 1990–2021 | -1.310 (-1.504 — -1.117) | <0.0001 | *** |
| DALYs | Global | Male | 80–84 years | 1990–2021 | -1.536 (-1.666 — -1.406) | <0.0001 | *** |
| DALYs | Global | Male | 85–89 years | 1990–2021 | -1.543 (-1.709 — -1.375) | <0.0001 | *** |
| DALYs | Global | Male | 90–94 years | 1990–2021 | -1.259 (-1.459 — -1.058) | <0.0001 | *** |
| DALYs | Global | Male | ≥95 years | 1990–2021 | -1.024 (-1.178 — -0.870) | <0.0001 | *** |
| DALYs | Global | Female | 70–74 years | 1990–2021 | -2.278 (-2.824 — -1.729) | <0.0001 | *** |
| DALYs | Global | Female | 75–79 years | 1990–2021 | -2.124 (-2.424 — -1.822) | <0.0001 | *** |
| DALYs | Global | Female | 80–84 years | 1990–2021 | -1.955 (-2.134 — -1.776) | <0.0001 | *** |
| DALYs | Global | Female | 85–89 years | 1990–2021 | -1.864 (-2.095 — -1.633) | <0.0001 | *** |
| DALYs | Global | Female | 90–94 years | 1990–2021 | -1.260 (-1.451 — -1.069) | <0.0001 | *** |
| DALYs | Global | Female | ≥95 years | 1990–2021 | -0.752 (-0.981 — -0.523) | <0.0001 | *** |
| DALYs | Global | Both | 70–74 years | 1990–2021 | -1.936 (-2.431 — -1.439) | <0.0001 | *** |
| DALYs | Global | Both | 75–79 years | 1990–2021 | -1.849 (-2.097 — -1.601) | <0.0001 | *** |
| DALYs | Global | Both | 80–84 years | 1990–2021 | -1.858 (-1.996 — -1.719) | <0.0001 | *** |
| DALYs | Global | Both | 85–89 years | 1990–2021 | -1.812 (-2.004 — -1.619) | <0.0001 | *** |
| DALYs | Global | Both | 90–94 years | 1990–2021 | -1.318 (-1.503 — -1.132) | <0.0001 | *** |
| DALYs | Global | Both | ≥95 years | 1990–2021 | -0.832 (-1.041 — -0.623) | <0.0001 | *** |
| DALYs | Low SDI | Male | 70–74 years | 1990–2021 | 2.546 (2.341 — 2.751) | <0.0001 | *** |
| DALYs | Low SDI | Male | 75–79 years | 1990–2021 | 3.047 (2.766 — 3.328) | <0.0001 | *** |
| DALYs | Low SDI | Male | 80–84 years | 1990–2021 | 3.191 (2.820 — 3.563) | <0.0001 | *** |
| DALYs | Low SDI | Male | 85–89 years | 1990–2021 | 3.764 (3.494 — 4.035) | <0.0001 | *** |
| DALYs | Low SDI | Male | 90–94 years | 1990–2021 | 3.843 (3.721 — 3.965) | <0.0001 | *** |
| DALYs | Low SDI | Male | ≥95 years | 1990–2021 | 3.704 (3.469 — 3.940) | <0.0001 | *** |
| DALYs | Low SDI | Female | 70–74 years | 1990–2021 | 0.982 (0.791 — 1.175) | <0.0001 | *** |
| DALYs | Low SDI | Female | 75–79 years | 1990–2021 | 1.460 (1.263 — 1.656) | <0.0001 | *** |
| DALYs | Low SDI | Female | 80–84 years | 1990–2021 | 1.869 (1.600 — 2.139) | <0.0001 | *** |
| DALYs | Low SDI | Female | 85–89 years | 1990–2021 | 2.359 (2.117 — 2.601) | <0.0001 | *** |
| DALYs | Low SDI | Female | 90–94 years | 1990–2021 | 1.962 (1.813 — 2.111) | <0.0001 | *** |
| DALYs | Low SDI | Female | ≥95 years | 1990–2021 | 1.617 (1.493 — 1.740) | <0.0001 | *** |
| DALYs | Low SDI | Both | 70–74 years | 1990–2021 | 1.543 (1.344 — 1.743) | <0.0001 | *** |
| DALYs | Low SDI | Both | 75–79 years | 1990–2021 | 1.975 (1.755 — 2.194) | <0.0001 | *** |
| DALYs | Low SDI | Both | 80–84 years | 1990–2021 | 2.276 (1.999 — 2.554) | <0.0001 | *** |
| DALYs | Low SDI | Both | 85–89 years | 1990–2021 | 2.751 (2.517 — 2.986) | <0.0001 | *** |
| DALYs | Low SDI | Both | 90–94 years | 1990–2021 | 2.402 (2.238 — 2.566) | <0.0001 | *** |
| DALYs | Low SDI | Both | ≥95 years | 1990–2021 | 2.153 (1.984 — 2.323) | <0.0001 | *** |
| DALYs | Low-middle SDI | Male | 70–74 years | 1990–2021 | 1.385 (1.277 — 1.493) | <0.0001 | *** |
| DALYs | Low-middle SDI | Male | 75–79 years | 1990–2021 | 1.555 (1.397 — 1.714) | <0.0001 | *** |
| DALYs | Low-middle SDI | Male | 80–84 years | 1990–2021 | 1.633 (1.487 — 1.780) | <0.0001 | *** |
| DALYs | Low-middle SDI | Male | 85–89 years | 1990–2021 | 2.049 (1.828 — 2.271) | <0.0001 | *** |
| DALYs | Low-middle SDI | Male | 90–94 years | 1990–2021 | 2.312 (1.904 — 2.721) | <0.0001 | *** |
| DALYs | Low-middle SDI | Male | ≥95 years | 1990–2021 | 2.415 (2.094 — 2.737) | <0.0001 | *** |
| DALYs | Low-middle SDI | Female | 70–74 years | 1990–2021 | 0.020 (-0.134 — 0.175) | 0.7956 | ns |
| DALYs | Low-middle SDI | Female | 75–79 years | 1990–2021 | 0.213 (0.099 — 0.328) | 0.0003 | *** |
| DALYs | Low-middle SDI | Female | 80–84 years | 1990–2021 | 0.591 (0.478 — 0.705) | <0.0001 | *** |
| DALYs | Low-middle SDI | Female | 85–89 years | 1990–2021 | 1.406 (1.217 — 1.596) | <0.0001 | *** |
| DALYs | Low-middle SDI | Female | 90–94 years | 1990–2021 | 1.674 (1.249 — 2.100) | <0.0001 | *** |
| DALYs | Low-middle SDI | Female | ≥95 years | 1990–2021 | 2.006 (1.436 — 2.579) | <0.0001 | *** |
| DALYs | Low-middle SDI | Both | 70–74 years | 1990–2021 | 0.552 (0.414 — 0.691) | <0.0001 | *** |
| DALYs | Low-middle SDI | Both | 75–79 years | 1990–2021 | 0.705 (0.591 — 0.819) | <0.0001 | *** |
| DALYs | Low-middle SDI | Both | 80–84 years | 1990–2021 | 0.981 (0.858 — 1.105) | <0.0001 | *** |
| DALYs | Low-middle SDI | Both | 85–89 years | 1990–2021 | 1.671 (1.475 — 1.868) | <0.0001 | *** |
| DALYs | Low-middle SDI | Both | 90–94 years | 1990–2021 | 1.925 (1.505 — 2.346) | <0.0001 | *** |
| DALYs | Low-middle SDI | Both | ≥95 years | 1990–2021 | 2.205 (1.706 — 2.705) | <0.0001 | *** |
| DALYs | Middle SDI | Male | 70–74 years | 1990–2021 | 0.679 (0.292 — 1.068) | 0.0006 | *** |
| DALYs | Middle SDI | Male | 75–79 years | 1990–2021 | 1.211 (0.969 — 1.452) | <0.0001 | *** |
| DALYs | Middle SDI | Male | 80–84 years | 1990–2021 | 0.849 (0.689 — 1.009) | <0.0001 | *** |
| DALYs | Middle SDI | Male | 85–89 years | 1990–2021 | 1.065 (0.916 — 1.215) | <0.0001 | *** |
| DALYs | Middle SDI | Male | 90–94 years | 1990–2021 | 0.962 (0.701 — 1.224) | <0.0001 | *** |
| DALYs | Middle SDI | Male | ≥95 years | 1990–2021 | 0.174 (-0.002 — 0.352) | 0.0533 | ns |
| DALYs | Middle SDI | Female | 70–74 years | 1990–2021 | 0.118 (0.017 — 0.219) | 0.0215 | * |
| DALYs | Middle SDI | Female | 75–79 years | 1990–2021 | 0.549 (0.407 — 0.690) | <0.0001 | *** |
| DALYs | Middle SDI | Female | 80–84 years | 1990–2021 | 0.186 (0.041 — 0.331) | 0.0120 | * |
| DALYs | Middle SDI | Female | 85–89 years | 1990–2021 | 0.101 (-0.048 — 0.251) | 0.1834 | ns |
| DALYs | Middle SDI | Female | 90–94 years | 1990–2021 | -0.089 (-0.210 — 0.032) | 0.1481 | ns |
| DALYs | Middle SDI | Female | ≥95 years | 1990–2021 | -0.073 (-0.318 — 0.172) | 0.5582 | ns |
| DALYs | Middle SDI | Both | 70–74 years | 1990–2021 | 0.351 (0.139 — 0.563) | 0.0012 | ** |
| DALYs | Middle SDI | Both | 75–79 years | 1990–2021 | 0.810 (0.631 — 0.989) | <0.0001 | *** |
| DALYs | Middle SDI | Both | 80–84 years | 1990–2021 | 0.429 (0.281 — 0.578) | <0.0001 | *** |
| DALYs | Middle SDI | Both | 85–89 years | 1990–2021 | 0.437 (0.309 — 0.564) | <0.0001 | *** |
| DALYs | Middle SDI | Both | 90–94 years | 1990–2021 | 0.217 (0.081 — 0.353) | 0.0017 | ** |
| DALYs | Middle SDI | Both | ≥95 years | 1990–2021 | -0.000 (-0.154 — 0.153) | 0.9963 | ns |
| DALYs | High-middle SDI | Male | 70–74 years | 1990–2021 | 0.254 (0.006 — 0.502) | 0.0448 | * |
| DALYs | High-middle SDI | Male | 75–79 years | 1990–2021 | 0.869 (0.466 — 1.273) | <0.0001 | *** |
| DALYs | High-middle SDI | Male | 80–84 years | 1990–2021 | 1.107 (0.742 — 1.474) | <0.0001 | *** |
| DALYs | High-middle SDI | Male | 85–89 years | 1990–2021 | 1.522 (1.160 — 1.885) | <0.0001 | *** |
| DALYs | High-middle SDI | Male | 90–94 years | 1990–2021 | 1.109 (0.897 — 1.320) | <0.0001 | *** |
| DALYs | High-middle SDI | Male | ≥95 years | 1990–2021 | 0.258 (0.114 — 0.401) | 0.0004 | *** |
| DALYs | High-middle SDI | Female | 70–74 years | 1990–2021 | -0.912 (-1.157 — -0.667) | <0.0001 | *** |
| DALYs | High-middle SDI | Female | 75–79 years | 1990–2021 | -0.195 (-0.557 — 0.169) | 0.2943 | ns |
| DALYs | High-middle SDI | Female | 80–84 years | 1990–2021 | 0.399 (-0.015 — 0.814) | 0.0591 | ns |
| DALYs | High-middle SDI | Female | 85–89 years | 1990–2021 | 0.778 (0.363 — 1.194) | 0.0002 | *** |
| DALYs | High-middle SDI | Female | 90–94 years | 1990–2021 | 0.596 (0.345 — 0.849) | <0.0001 | *** |
| DALYs | High-middle SDI | Female | ≥95 years | 1990–2021 | 0.409 (0.313 — 0.505) | <0.0001 | *** |
| DALYs | High-middle SDI | Both | 70–74 years | 1990–2021 | -0.422 (-0.654 — -0.189) | 0.0004 | *** |
| DALYs | High-middle SDI | Both | 75–79 years | 1990–2021 | 0.188 (-0.177 — 0.553) | 0.3135 | ns |
| DALYs | High-middle SDI | Both | 80–84 years | 1990–2021 | 0.605 (0.220 — 0.992) | 0.0020 | ** |
| DALYs | High-middle SDI | Both | 85–89 years | 1990–2021 | 1.001 (0.614 — 1.390) | <0.0001 | *** |
| DALYs | High-middle SDI | Both | 90–94 years | 1990–2021 | 0.705 (0.483 — 0.927) | <0.0001 | *** |
| DALYs | High-middle SDI | Both | ≥95 years | 1990–2021 | 0.359 (0.278 — 0.439) | <0.0001 | *** |
| DALYs | High SDI | Male | 70–74 years | 1990–2021 | -2.321 (-2.858 — -1.782) | <0.0001 | *** |
| DALYs | High SDI | Male | 75–79 years | 1990–2021 | -2.054 (-2.251 — -1.856) | <0.0001 | *** |
| DALYs | High SDI | Male | 80–84 years | 1990–2021 | -2.170 (-2.295 — -2.044) | <0.0001 | *** |
| DALYs | High SDI | Male | 85–89 years | 1990–2021 | -2.155 (-2.336 — -1.974) | <0.0001 | *** |
| DALYs | High SDI | Male | 90–94 years | 1990–2021 | -1.735 (-1.950 — -1.519) | <0.0001 | *** |
| DALYs | High SDI | Male | ≥95 years | 1990–2021 | -1.175 (-1.392 — -0.957) | <0.0001 | *** |
| DALYs | High SDI | Female | 70–74 years | 1990–2021 | -3.209 (-3.988 — -2.423) | <0.0001 | *** |
| DALYs | High SDI | Female | 75–79 years | 1990–2021 | -2.771 (-3.185 — -2.354) | <0.0001 | *** |
| DALYs | High SDI | Female | 80–84 years | 1990–2021 | -2.346 (-2.581 — -2.110) | <0.0001 | *** |
| DALYs | High SDI | Female | 85–89 years | 1990–2021 | -2.074 (-2.327 — -1.820) | <0.0001 | *** |
| DALYs | High SDI | Female | 90–94 years | 1990–2021 | -1.293 (-1.509 — -1.076) | <0.0001 | *** |
| DALYs | High SDI | Female | ≥95 years | 1990–2021 | -0.668 (-0.931 — -0.404) | <0.0001 | *** |
| DALYs | High SDI | Both | 70–74 years | 1990–2021 | -2.804 (-3.471 — -2.131) | <0.0001 | *** |
| DALYs | High SDI | Both | 75–79 years | 1990–2021 | -2.517 (-2.841 — -2.193) | <0.0001 | *** |
| DALYs | High SDI | Both | 80–84 years | 1990–2021 | -2.320 (-2.504 — -2.136) | <0.0001 | *** |
| DALYs | High SDI | Both | 85–89 years | 1990–2021 | -2.133 (-2.351 — -1.914) | <0.0001 | *** |
| DALYs | High SDI | Both | 90–94 years | 1990–2021 | -1.445 (-1.661 — -1.229) | <0.0001 | *** |
| DALYs | High SDI | Both | ≥95 years | 1990–2021 | -0.784 (-1.039 — -0.529) | <0.0001 | *** |

APCs were estimated from log-linear regression with HAC standard errors (maxlag = 1). APC = (exp(β) − 1) × 100. * p < 0.05, ** p < 0.01, *** p < 0.001..

**Supplementary Table S6.** Period rate ratios (PRRs) by 5-year period, SDI level, and sex

| **Measure** | **Location** | **Sex** | **Reference period** | **Period** | **Reference rate mean** | **Period rate mean** | **PRR** |
| --- | --- | --- | --- | --- | --- | --- | --- |
| DALYs | Global | Male | 1990–1994 | 1990–1994 | 372.257 | 372.257 | 1.000 |
| DALYs | Global | Male | 1990–1994 | 1995–1999 | 372.257 | 373.044 | 1.002 |
| DALYs | Global | Male | 1990–1994 | 2000–2004 | 372.257 | 362.855 | 0.975 |
| DALYs | Global | Male | 1990–1994 | 2005–2009 | 372.257 | 323.041 | 0.868 |
| DALYs | Global | Male | 1990–1994 | 2010–2014 | 372.257 | 290.422 | 0.780 |
| DALYs | Global | Male | 1990–1994 | 2015–2019 | 372.257 | 269.471 | 0.724 |
| DALYs | Global | Male | 1990–1994 | 2020–2021 | 372.257 | 259.063 | 0.696 |
| DALYs | Global | Female | 1990–1994 | 1990–1994 | 490.146 | 490.146 | 1.000 |
| DALYs | Global | Female | 1990–1994 | 1995–1999 | 490.146 | 493.650 | 1.007 |
| DALYs | Global | Female | 1990–1994 | 2000–2004 | 490.146 | 473.255 | 0.966 |
| DALYs | Global | Female | 1990–1994 | 2005–2009 | 490.146 | 403.333 | 0.823 |
| DALYs | Global | Female | 1990–1994 | 2010–2014 | 490.146 | 346.646 | 0.707 |
| DALYs | Global | Female | 1990–1994 | 2015–2019 | 490.146 | 311.728 | 0.636 |
| DALYs | Global | Female | 1990–1994 | 2020–2021 | 490.146 | 289.598 | 0.591 |
| DALYs | Global | Both | 1990–1994 | 1990–1994 | 441.937 | 441.937 | 1.000 |
| DALYs | Global | Both | 1990–1994 | 1995–1999 | 441.937 | 443.607 | 1.004 |
| DALYs | Global | Both | 1990–1994 | 2000–2004 | 441.937 | 426.468 | 0.965 |
| DALYs | Global | Both | 1990–1994 | 2005–2009 | 441.937 | 368.867 | 0.835 |
| DALYs | Global | Both | 1990–1994 | 2010–2014 | 441.937 | 322.303 | 0.729 |
| DALYs | Global | Both | 1990–1994 | 2015–2019 | 441.937 | 293.213 | 0.663 |
| DALYs | Global | Both | 1990–1994 | 2020–2021 | 441.937 | 276.123 | 0.625 |
| DALYs | Low SDI | Male | 1990–1994 | 1990–1994 | 57.823 | 57.823 | 1.000 |
| DALYs | Low SDI | Male | 1990–1994 | 1995–1999 | 57.823 | 66.215 | 1.145 |
| DALYs | Low SDI | Male | 1990–1994 | 2000–2004 | 57.823 | 76.805 | 1.328 |
| DALYs | Low SDI | Male | 1990–1994 | 2005–2009 | 57.823 | 90.850 | 1.571 |
| DALYs | Low SDI | Male | 1990–1994 | 2010–2014 | 57.823 | 106.982 | 1.850 |
| DALYs | Low SDI | Male | 1990–1994 | 2015–2019 | 57.823 | 113.772 | 1.968 |
| DALYs | Low SDI | Male | 1990–1994 | 2020–2021 | 57.823 | 118.102 | 2.042 |
| DALYs | Low SDI | Female | 1990–1994 | 1990–1994 | 165.172 | 165.172 | 1.000 |
| DALYs | Low SDI | Female | 1990–1994 | 1995–1999 | 165.172 | 173.009 | 1.047 |
| DALYs | Low SDI | Female | 1990–1994 | 2000–2004 | 165.172 | 188.193 | 1.139 |
| DALYs | Low SDI | Female | 1990–1994 | 2005–2009 | 165.172 | 206.348 | 1.249 |
| DALYs | Low SDI | Female | 1990–1994 | 2010–2014 | 165.172 | 223.880 | 1.355 |
| DALYs | Low SDI | Female | 1990–1994 | 2015–2019 | 165.172 | 227.243 | 1.376 |
| DALYs | Low SDI | Female | 1990–1994 | 2020–2021 | 165.172 | 220.503 | 1.335 |
| DALYs | Low SDI | Both | 1990–1994 | 1990–1994 | 111.293 | 111.293 | 1.000 |
| DALYs | Low SDI | Both | 1990–1994 | 1995–1999 | 111.293 | 119.430 | 1.073 |
| DALYs | Low SDI | Both | 1990–1994 | 2000–2004 | 111.293 | 132.513 | 1.191 |
| DALYs | Low SDI | Both | 1990–1994 | 2005–2009 | 111.293 | 149.596 | 1.344 |
| DALYs | Low SDI | Both | 1990–1994 | 2010–2014 | 111.293 | 167.304 | 1.503 |
| DALYs | Low SDI | Both | 1990–1994 | 2015–2019 | 111.293 | 172.424 | 1.549 |
| DALYs | Low SDI | Both | 1990–1994 | 2020–2021 | 111.293 | 171.256 | 1.539 |
| DALYs | Low-middle SDI | Male | 1990–1994 | 1990–1994 | 117.519 | 117.519 | 1.000 |
| DALYs | Low-middle SDI | Male | 1990–1994 | 1995–1999 | 117.519 | 123.432 | 1.050 |
| DALYs | Low-middle SDI | Male | 1990–1994 | 2000–2004 | 117.519 | 130.355 | 1.109 |
| DALYs | Low-middle SDI | Male | 1990–1994 | 2005–2009 | 117.519 | 141.133 | 1.201 |
| DALYs | Low-middle SDI | Male | 1990–1994 | 2010–2014 | 117.519 | 157.017 | 1.336 |
| DALYs | Low-middle SDI | Male | 1990–1994 | 2015–2019 | 117.519 | 169.063 | 1.439 |
| DALYs | Low-middle SDI | Male | 1990–1994 | 2020–2021 | 117.519 | 172.881 | 1.471 |
| DALYs | Low-middle SDI | Female | 1990–1994 | 1990–1994 | 246.208 | 246.208 | 1.000 |
| DALYs | Low-middle SDI | Female | 1990–1994 | 1995–1999 | 246.208 | 241.274 | 0.980 |
| DALYs | Low-middle SDI | Female | 1990–1994 | 2000–2004 | 246.208 | 239.308 | 0.972 |
| DALYs | Low-middle SDI | Female | 1990–1994 | 2005–2009 | 246.208 | 242.438 | 0.985 |
| DALYs | Low-middle SDI | Female | 1990–1994 | 2010–2014 | 246.208 | 247.940 | 1.007 |
| DALYs | Low-middle SDI | Female | 1990–1994 | 2015–2019 | 246.208 | 258.632 | 1.050 |
| DALYs | Low-middle SDI | Female | 1990–1994 | 2020–2021 | 246.208 | 263.341 | 1.070 |
| DALYs | Low-middle SDI | Both | 1990–1994 | 1990–1994 | 183.919 | 183.919 | 1.000 |
| DALYs | Low-middle SDI | Both | 1990–1994 | 1995–1999 | 183.919 | 184.233 | 1.002 |
| DALYs | Low-middle SDI | Both | 1990–1994 | 2000–2004 | 183.919 | 186.904 | 1.016 |
| DALYs | Low-middle SDI | Both | 1990–1994 | 2005–2009 | 183.919 | 194.901 | 1.060 |
| DALYs | Low-middle SDI | Both | 1990–1994 | 2010–2014 | 183.919 | 206.018 | 1.120 |
| DALYs | Low-middle SDI | Both | 1990–1994 | 2015–2019 | 183.919 | 217.437 | 1.182 |
| DALYs | Low-middle SDI | Both | 1990–1994 | 2020–2021 | 183.919 | 221.893 | 1.206 |
| DALYs | Middle SDI | Male | 1990–1994 | 1990–1994 | 252.691 | 252.691 | 1.000 |
| DALYs | Middle SDI | Male | 1990–1994 | 1995–1999 | 252.691 | 277.354 | 1.098 |
| DALYs | Middle SDI | Male | 1990–1994 | 2000–2004 | 252.691 | 305.264 | 1.208 |
| DALYs | Middle SDI | Male | 1990–1994 | 2005–2009 | 252.691 | 309.022 | 1.223 |
| DALYs | Middle SDI | Male | 1990–1994 | 2010–2014 | 252.691 | 315.177 | 1.247 |
| DALYs | Middle SDI | Male | 1990–1994 | 2015–2019 | 252.691 | 318.526 | 1.261 |
| DALYs | Middle SDI | Male | 1990–1994 | 2020–2021 | 252.691 | 318.640 | 1.261 |
| DALYs | Middle SDI | Female | 1990–1994 | 1990–1994 | 327.837 | 327.837 | 1.000 |
| DALYs | Middle SDI | Female | 1990–1994 | 1995–1999 | 327.837 | 329.582 | 1.005 |
| DALYs | Middle SDI | Female | 1990–1994 | 2000–2004 | 327.837 | 346.337 | 1.056 |
| DALYs | Middle SDI | Female | 1990–1994 | 2005–2009 | 327.837 | 350.979 | 1.071 |
| DALYs | Middle SDI | Female | 1990–1994 | 2010–2014 | 327.837 | 344.657 | 1.051 |
| DALYs | Middle SDI | Female | 1990–1994 | 2015–2019 | 327.837 | 343.844 | 1.049 |
| DALYs | Middle SDI | Female | 1990–1994 | 2020–2021 | 327.837 | 342.948 | 1.046 |
| DALYs | Middle SDI | Both | 1990–1994 | 1990–1994 | 294.739 | 294.739 | 1.000 |
| DALYs | Middle SDI | Both | 1990–1994 | 1995–1999 | 294.739 | 306.589 | 1.040 |
| DALYs | Middle SDI | Both | 1990–1994 | 2000–2004 | 294.739 | 328.187 | 1.113 |
| DALYs | Middle SDI | Both | 1990–1994 | 2005–2009 | 294.739 | 332.523 | 1.128 |
| DALYs | Middle SDI | Both | 1990–1994 | 2010–2014 | 294.739 | 331.731 | 1.126 |
| DALYs | Middle SDI | Both | 1990–1994 | 2015–2019 | 294.739 | 332.715 | 1.129 |
| DALYs | Middle SDI | Both | 1990–1994 | 2020–2021 | 294.739 | 332.213 | 1.127 |
| DALYs | High-middle SDI | Male | 1990–1994 | 1990–1994 | 175.681 | 175.681 | 1.000 |
| DALYs | High-middle SDI | Male | 1990–1994 | 1995–1999 | 175.681 | 190.668 | 1.085 |
| DALYs | High-middle SDI | Male | 1990–1994 | 2000–2004 | 175.681 | 203.186 | 1.157 |
| DALYs | High-middle SDI | Male | 1990–1994 | 2005–2009 | 175.681 | 206.502 | 1.175 |
| DALYs | High-middle SDI | Male | 1990–1994 | 2010–2014 | 175.681 | 211.211 | 1.202 |
| DALYs | High-middle SDI | Male | 1990–1994 | 2015–2019 | 175.681 | 209.355 | 1.192 |
| DALYs | High-middle SDI | Male | 1990–1994 | 2020–2021 | 175.681 | 193.509 | 1.101 |
| DALYs | High-middle SDI | Female | 1990–1994 | 1990–1994 | 248.882 | 248.882 | 1.000 |
| DALYs | High-middle SDI | Female | 1990–1994 | 1995–1999 | 248.882 | 261.792 | 1.052 |
| DALYs | High-middle SDI | Female | 1990–1994 | 2000–2004 | 248.882 | 268.963 | 1.081 |
| DALYs | High-middle SDI | Female | 1990–1994 | 2005–2009 | 248.882 | 254.179 | 1.021 |
| DALYs | High-middle SDI | Female | 1990–1994 | 2010–2014 | 248.882 | 248.061 | 0.997 |
| DALYs | High-middle SDI | Female | 1990–1994 | 2015–2019 | 248.882 | 238.583 | 0.959 |
| DALYs | High-middle SDI | Female | 1990–1994 | 2020–2021 | 248.882 | 212.219 | 0.853 |
| DALYs | High-middle SDI | Both | 1990–1994 | 1990–1994 | 216.484 | 216.484 | 1.000 |
| DALYs | High-middle SDI | Both | 1990–1994 | 1995–1999 | 216.484 | 229.828 | 1.062 |
| DALYs | High-middle SDI | Both | 1990–1994 | 2000–2004 | 216.484 | 238.950 | 1.104 |
| DALYs | High-middle SDI | Both | 1990–1994 | 2005–2009 | 216.484 | 232.344 | 1.073 |
| DALYs | High-middle SDI | Both | 1990–1994 | 2010–2014 | 216.484 | 231.171 | 1.068 |
| DALYs | High-middle SDI | Both | 1990–1994 | 2015–2019 | 216.484 | 225.157 | 1.040 |
| DALYs | High-middle SDI | Both | 1990–1994 | 2020–2021 | 216.484 | 203.628 | 0.941 |
| DALYs | High SDI | Male | 1990–1994 | 1990–1994 | 547.735 | 547.735 | 1.000 |
| DALYs | High SDI | Male | 1990–1994 | 1995–1999 | 547.735 | 540.275 | 0.986 |
| DALYs | High SDI | Male | 1990–1994 | 2000–2004 | 547.735 | 513.298 | 0.937 |
| DALYs | High SDI | Male | 1990–1994 | 2005–2009 | 547.735 | 436.847 | 0.798 |
| DALYs | High SDI | Male | 1990–1994 | 2010–2014 | 547.735 | 370.643 | 0.677 |
| DALYs | High SDI | Male | 1990–1994 | 2015–2019 | 547.735 | 330.815 | 0.604 |
| DALYs | High SDI | Male | 1990–1994 | 2020–2021 | 547.735 | 315.818 | 0.577 |
| DALYs | High SDI | Female | 1990–1994 | 1990–1994 | 623.344 | 623.344 | 1.000 |
| DALYs | High SDI | Female | 1990–1994 | 1995–1999 | 623.344 | 630.156 | 1.011 |
| DALYs | High SDI | Female | 1990–1994 | 2000–2004 | 623.344 | 601.198 | 0.964 |
| DALYs | High SDI | Female | 1990–1994 | 2005–2009 | 623.344 | 497.151 | 0.798 |
| DALYs | High SDI | Female | 1990–1994 | 2010–2014 | 623.344 | 407.107 | 0.653 |
| DALYs | High SDI | Female | 1990–1994 | 2015–2019 | 623.344 | 351.610 | 0.564 |
| DALYs | High SDI | Female | 1990–1994 | 2020–2021 | 623.344 | 323.428 | 0.519 |
| DALYs | High SDI | Both | 1990–1994 | 1990–1994 | 595.045 | 595.045 | 1.000 |
| DALYs | High SDI | Both | 1990–1994 | 1995–1999 | 595.045 | 595.851 | 1.001 |
| DALYs | High SDI | Both | 1990–1994 | 2000–2004 | 595.045 | 566.589 | 0.952 |
| DALYs | High SDI | Both | 1990–1994 | 2005–2009 | 595.045 | 472.786 | 0.795 |
| DALYs | High SDI | Both | 1990–1994 | 2010–2014 | 595.045 | 392.094 | 0.659 |
| DALYs | High SDI | Both | 1990–1994 | 2015–2019 | 595.045 | 342.888 | 0.576 |
| DALYs | High SDI | Both | 1990–1994 | 2020–2021 | 595.045 | 320.189 | 0.538 |

PRR, period rate ratio; 1990–1994 was the reference period.

**Supplementary Table S7.** Hindcast performance metrics by outcome and SDI level, including MAPE, MAE, RMSE, and 95% prediction-interval coverage

| **Measure** | **Location** | **MAPE (%)** | **MAE** | **RMSE** | **Coverage within 95% PI (%)** |
| --- | --- | --- | --- | --- | --- |
| Deaths | Global | 2.98 | 0.53 | 0.55 | 100.00 |
| Deaths | Low SDI | 7.58 | 0.74 | 0.87 | 28.57 |
| Deaths | Low-middle SDI | 0.75 | 0.10 | 0.11 | 85.71 |
| Deaths | Middle SDI | 1.39 | 0.27 | 0.33 | 85.71 |
| Deaths | High-middle SDI | 6.63 | 0.79 | 1.07 | 57.14 |
| Deaths | High SDI | 4.40 | 0.92 | 0.94 | 100.00 |
| DALYs | Global | 5.10 | 14.54 | 15.33 | 100.00 |
| DALYs | Low SDI | 7.28 | 12.47 | 14.63 | 28.57 |
| DALYs | Low-middle SDI | 0.93 | 2.04 | 2.27 | 85.71 |
| DALYs | Middle SDI | 1.49 | 5.00 | 6.38 | 85.71 |
| DALYs | High-middle SDI | 4.04 | 8.47 | 11.05 | 100.00 |
| DALYs | High SDI | 7.04 | 23.29 | 25.00 | 100.00 |

**Supplementary Table S8.** Summary of multi-model robustness comparisons for the global aggregated ≥70-year death-rate series

| **Sex** | **Primary model** | **Alternative model** | **MAPD, 2022–2044 (%)** | **2044 rate (primary)** | **2044 rate (alternative)** | **Difference in 2044 rate vs primary (%)** |
| --- | --- | --- | --- | --- | --- | --- |
| Both | log-linear | UCM | 3.44 | 9.578 | 10.043 | 4.86 |
| Both | log-linear | ARIMA | 9.21 | 9.578 | 11.317 | 18.15 |
| Male | log-linear | UCM | 3.69 | 9.383 | 9.860 | 5.09 |
| Male | log-linear | ARIMA | 10.55 | 9.383 | 11.326 | 20.71 |
| Female | log-linear | UCM | 3.10 | 9.996 | 10.447 | 4.52 |
| Female | log-linear | ARIMA | 7.63 | 9.996 | 11.495 | 15.00 |
